# Istradefylline protects from cisplatin-induced nephrotoxicity and peripheral neuropathy while preserving cisplatin antitumor effects

**DOI:** 10.1172/JCI152924

**Published:** 2022-11-15

**Authors:** Edmone Dewaeles, Kévin Carvalho, Sandy Fellah, Jaewon Sim, Nihad Boukrout, Raphaelle Caillierez, Hariharan Ramakrishnan, Cynthia Van der Hauwaert, Jhenkruthi Vijaya Shankara, Nathalie Martin, Noura Massri, Agathe Launay, Joseph K. Folger, Clémentine de Schutter, Romain Larrue, Ingrid Loison, Marine Goujon, Matthieu Jung, Stéphanie Le Gras, Victoria Gomez-Murcia, Emilie Faivre, Julie Lemaire, Anne Garat, Nicolas Beauval, Patrice Maboudou, Viviane Gnemmi, Jean-Baptiste Gibier, Luc Buée, Corinne Abbadie, Francois Glowacki, Nicolas Pottier, Michael Perrais, Rodrigo A. Cunha, Jean-Sébastien Annicotte, Geoffroy Laumet, David Blum, Christelle Cauffiez

**Affiliations:** 1 University of Lille, INSERM, CNRS, CHU Lille, UMR9020-U1277, CANTHER, Cancer Heterogeneity, Plasticity and Resistance to Therapies, Lille, France.; 2 University of Lille, INSERM, CHU Lille, UMR-S1172 LilNCog, Lille Neuroscience and Cognition, Lille, France.; 3 Alzheimer and Tauopathies, LabEx DISTALZ, Lille, France.; 4 Department of Physiology, Michigan State University, East Lansing, Michigan, USA.; 5 Cell and Molecular Biology Graduate program, Michigan State University, East Lansing, Michigan, USA.; 6 CHU Lille, Département de la Recherche en Santé, Lille, France.; 7 CHU Lille, Service de Toxicologie et Génopathies, Lille, France.; 8 University of Strasbourg, CNRS UMR 7104, INSERM U1258 – GenomEast Platform – IGBMC – Institut de Génétique et de Biologie Moléculaire et Cellulaire, Illkirch, France.; 9 University of Lille, CHU Lille, Institut Pasteur de Lille, ULR 4483, IMPact de l’Environnement Chimique sur la Santé Humaine (IMPECS), Lille, France.; 10 CHU Lille, Service de Biochimie Automatisée, Protéines et Biologie Prédictive, Lille, France.; 11 CHU Lille, Service d’Anatomopathologie, Lille, France.; 12 CHU Lille, Service de Néphrologie, Lille, France.; 13 CNC, Center for Neuroscience and Cell Biology, University of Coimbra, Rua Larga, Faculty of Medicine Building-Polo 1, Coimbra, Portugal.; 14 Faculty of Medicine, University of Coimbra, Coimbra, Portugal.; 15 University of Lille, INSERM, CNRS, CHU Lille, Institut Pasteur de Lille, INSERM U1283-UMR8199 – EGID, Lille, France.; 16 University of Lille, INSERM, CHU Lille, Institut Pasteur de Lille, RID-AGE-Facteurs de risque et déterminants moléculaires des maladies liées au vieillissement, Lille, France.

**Keywords:** Nephrology, G protein&ndash;coupled receptors, Pharmacology

## Abstract

Cisplatin is a potent chemotherapeutic drug that is widely used in the treatment of various solid cancers. However, its clinical effectiveness is strongly limited by frequent severe adverse effects, in particular nephrotoxicity and chemotherapy-induced peripheral neuropathy. Thus, there is an urgent medical need to identify novel strategies that limit cisplatin-induced toxicity. In the present study, we show that the FDA-approved adenosine A_2A_ receptor antagonist istradefylline (KW6002) protected from cisplatin-induced nephrotoxicity and neuropathic pain in mice with or without tumors. Moreover, we also demonstrate that the antitumoral properties of cisplatin were not altered by istradefylline in tumor-bearing mice and could even be potentiated. Altogether, our results support the use of istradefylline as a valuable preventive approach for the clinical management of patients undergoing cisplatin treatment.

## Graphical abstract

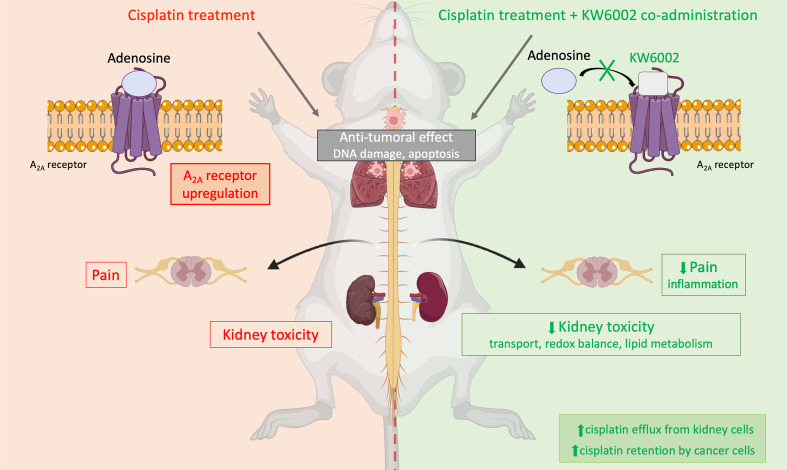

## Introduction

Cisplatin is a potent antineoplastic agent that is widely used in the treatment of various solid cancers such as lung, ovarian, and testicular cancers as well as HPV^+^ squamous carcinoma (1, 2). The antitumor action of cisplatin requires its intracellular bioactivation by the replacement of chlorides with water molecules, forming a highly reactive molecule that binds to DNA and induces cytotoxic lesions in tumors (3). However, the unwanted accumulation of cisplatin in healthy cells can also trigger cytotoxicity. Indeed, the clinical use of cisplatin is restricted by various severe adverse effects, including nephrotoxicity and chemotherapy-induced peripheral neuropathy (CIPN) (4–6). In the kidney, cisplatin promotes primarily proximal tubular cell injury and death through several pathways, including apoptosis (7). The antitumor properties as well as the side effects of cisplatin are both dependent on its intracellular accumulation, which is mediated, at least in part, by membrane transporters (8). Renal toxicity of cisplatin is cumulative and dose dependent, leading to tubular lesions associated with a lower glomerular filtration rate (9, 10). Cisplatin has also been reported to induce acute renal failure in up to 35 % of patients, leading to cisplatin dose adjustments or even withdrawal, thereby adversely affecting patient outcomes (11, 12).

In clinical practice, the prevention of cisplatin-induced nephrotoxicity still largely relies on nonspecific interventions, such as saline hydration or magnesium infusion (4, 12). Similarly, CIPN is often considered a frequent but unavoidable adverse effect of cisplatin chemotherapy that should be accepted by patients (13). Therefore, there is an urgent medical need for strategies that alleviate cisplatin-induced nephrotoxicity and peripheral neuropathy, without interfering with the efficiency of cisplatin to control tumor growth.

Adenosine plays a major role in cellular and tissue homeostasis (14–16). Its physiological function relies on 4 GPCRs: A_1_, A_2A_, A_2B_, and A_3_ (17–20). Adenosine is important for several aspects of renal physiology (21, 22), and adenosine and its receptors are involved in various types of kidney injuries (23–27). In particular, the pharmacological blockade of A_1_ receptors using several antagonists, such as tonapofylline (28), 8-cyclopentyl-1,3-dipropylxanthine (29), or KW-3902 (30), has been reported to offer protection against cisplatin nephrotoxicity in rodent models. The adenosine A_2A_ receptor (A_2A_R) also controls renal pathologies of various etiologies such as ischemia-reperfusion injury (31, 32), fibrosis (26, 33), diabetic nephropathy (34), and glomerulonephritis (35). However, the role of the A_2A_R still remains unclear in the context of cisplatin-induced toxicity. In the present study, using mouse models of acute, subchronic, and cumulative chronic cisplatin administration, we serendipitously observed that istradefylline (KW6002), an A_2A_R antagonist, mitigated cisplatin-induced nephrotoxicity and pain hypersensitivity, and did not decrease, but rather potentiated, the antitumoral properties of cisplatin. Importantly, KW6002 has been FDA approved as an add-on treatment to levodopa in the treatment of patients with Parkinson’s disease with OFF episodes (36, 37). These data support the repurposing of istradefylline as a valuable preventive approach for patients undergoing cisplatin treatment.

## Results

### Cisplatin-induced nephrotoxicity is associated with renal A_2A_R upregulation in mice.

**Figure 1:**
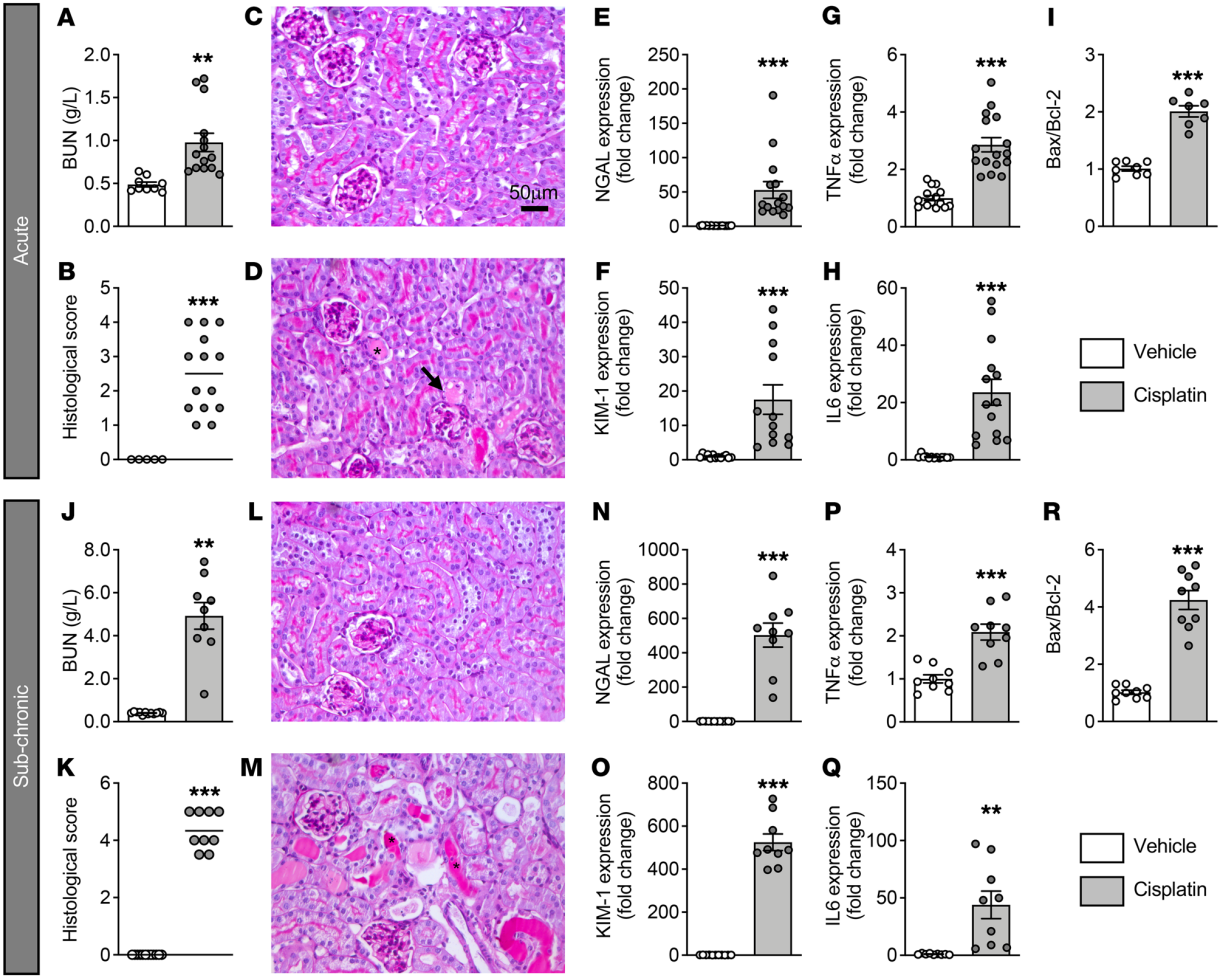
Kidney injury induced by acute and subchronic administration of cisplatin. Results in **A**–**I** correspond to kidney injury induced by acute administration of cisplatin, and results in **J**–**R** correspond to kidney injury induced by subchronic administration of cisplatin. (**A** and **J**) BUN quantification. (**B** and **K**). Renal injury histological scores. (**C**, **D**, **L**, and **M**) Representative images of periodic acid–Schiff–stained kidney sections from vehicle-treated (**C** and **L**) and cisplatin-treated (**D** and **M**) mice. Asterisks indicate tubular casts. Arrow indicates necrosis. Scale bar: 50 μm. (**E**, **F**, **N**, and **O**) Gene expression of the renal injury markers *NGAL* (**E** and **N**) and *KIM1* (**F** and **O**). Gene expression of the inflammatory markers *Tnfa* (**G** and **P**) and *Il6* (**H** and **Q**). Gene expression ratio of the apoptotic markers *Bax* and *Bcl2* (**I** and **R**). Results indicate the mean ± SEM. **P < 0.01 and ***P < 0.001; 2-tailed, unpaired Student’s *t* test (*n* = 8–16 animals/group.

Mice treated with cisplatin either acutely (acute A model: a single dose of 10 mg/kg; Supplemental Figure 1A; supplemental material available online with this article; https://doi.org/10.1172/JCI152924DS1) or subchronically (subchronic [SC] model: 3 mg/kg for 6 days; Supplemental Figure 1B) exhibited marked renal dysfunction, as shown by increased blood urea nitrogen (BUN) levels (Figure 1, A and J) as well as severe histological lesions (Figure 1, B–D and K–M), including the presence of necrotic cells and tubular casts. Accordingly, mRNA levels of 2 renal injury markers, neutrophil gelatinase–associated lipocalin (*NGAL*) and kidney injury molecule 1 (*KIM1*), were significantly increased (Figure 1, E, F, N, and O). Cisplatin nephrotoxicity was associated with an inflammatory response and apoptosis, as indicated by the increased mRNA expression of *Il6* and *Tnfa* (Figure 1, G, H, P, and Q) and the enhanced Bcl2-associated X/B cell lymphoma 2 (*Bax/Bcl2*) ratio (Figure 1, I and R), as previously described (38).

**Figure 2:**
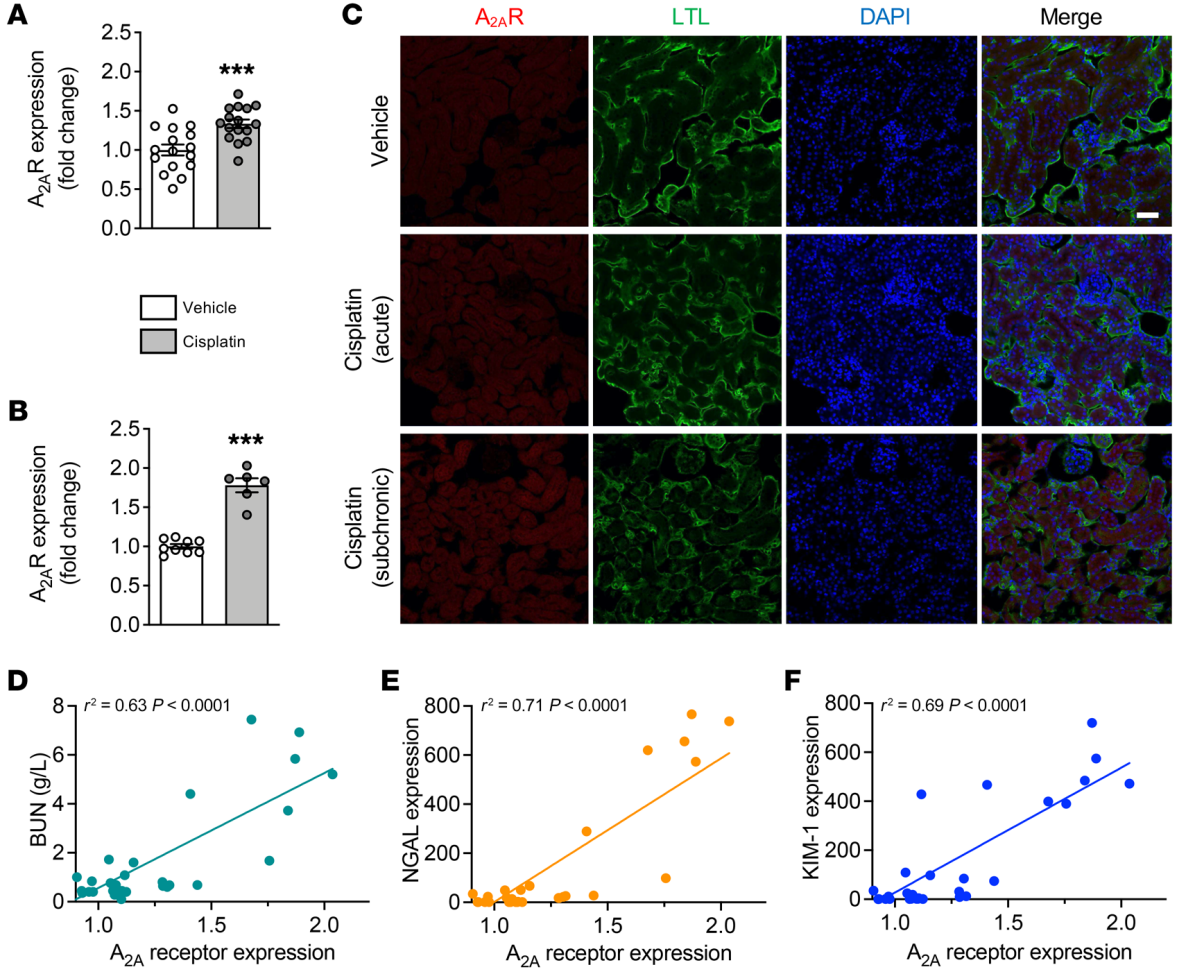
A_2A_R upregulation in acute and subchronic models of cisplatin-induced nephrotoxicity. (**A** and **B**) Renal *A_2A_R* levels following acute (**A**) or subchronic (**B**) cisplatin administration. Results are the mean ± SEM. ***P < 0.001 versus vehicle; 2-tailed, unpaired Student’s *t* test (*n* = 6–16 animals/group). (**C**) Representative images of the immunofluorescence staining for A_2A_R in renal samples from animals treated with vehicle or acute or subchronic cisplatin administration. Kidney sections were stained with the proximal tubule marker reagent *Lotus tetragonolobus* lectin (LTL). Scale bar: 50 μm. (**D**–**F**) Linear correlation between *A_2A_R* mRNA levels and the renal injury markers BUN (**D**), *NGAL* (**E**), and *KIM1* (**F**).

Interestingly, we observed that cisplatin promoted the upregulation of A_2A_R (Figure 2, A–C). Immunofluorescence showed that, the A_2A_R was expressed in renal cells, and especially in epithelial tubular cells (Figure 2C). Furthermore, A_2A_R levels were significantly correlated with BUN levels (*r^2^* = 0.63, *P* < 0.0001) as well as with *NGAL* (*r^2^* = 0.71, *P* < 0.0001) and *KIM1* (*r^2^* = 0.69, *P* < 0.0001) expression (Figure 2, D–F). These data suggest that A_2A_R dysregulation might be associated with the pathological processes underlying cisplatin-induced nephrotoxicity.

### A_2A_R antagonism alleviates cisplatin-induced toxicity in vivo.

**Figure 3:**
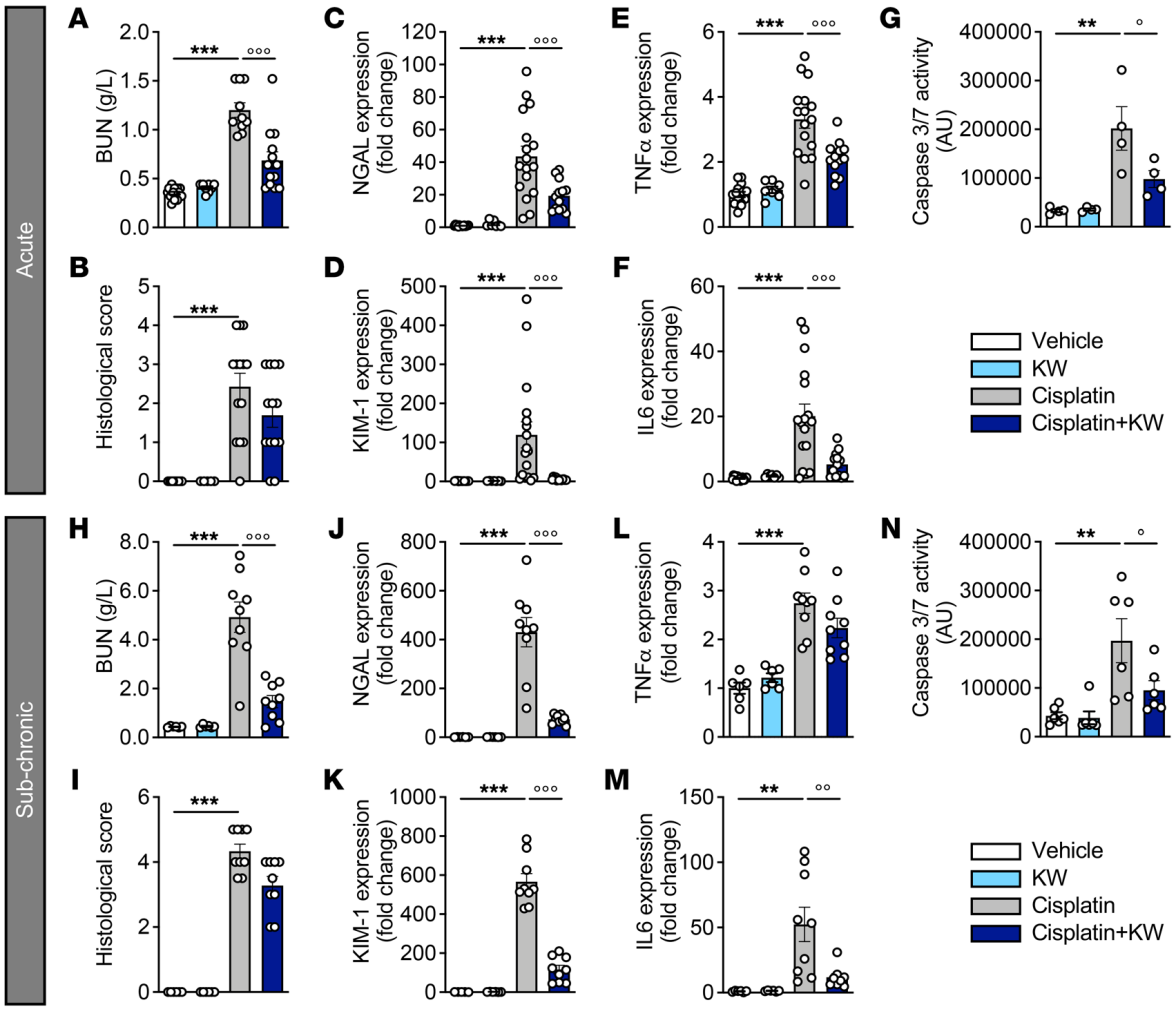
KW6002 protects against cisplatin-induced kidney injury in acute and subchronic models. Results in **A**–**G** correspond to the acute model and results in **H**–**N** to the subchronic model. (**A** and **H**) BUN quantification. (**B** and **I**) Renal injury histological scores. Gene expression of the renal injury markers *NGAL* (**C** and **J**) and *KIM1* (**D** and **K**). Gene expression of the inflammatory markers *Tnfa* (**E** and **L**) and *Il6* (**F** and **M**). Caspase 3/-7 activity (**G** and **N**). Results are the mean ± SEM. **P < 0.01 and ***P < 0.001 versus vehicle; °*P* < 0.05, °°*P* < 0.01, and °°°*P* < 0.001 versus cisplatin; 1-way ANOVA followed by Tukey’s post hoc test (*n* = 4–17 animals/group). KW, KW6002.

To assess whether A_2A_R function is involved in cisplatin-induced renal injury, we evaluated the impact of the FDA-approved selective A_2A_R antagonist istradefylline (KW6002) in both acute (A) and subchronic (SC) models of cisplatin-induced kidney toxicity (Supplemental Figure 1, C and D). While not significantly impacting kidney histology (Figure 3, B and I), KW6002 mitigated renal dysfunction induced by cisplatin, as shown by the significant reduction in BUN levels (A model: –42.9% ± 7.4%; SC model: –70.2% ± 5.1% vs. cisplatin; Figure 3, A and H), *NGAL* (A: –55.5% ± 5.9%; SC: –82.9% ± 1.4% vs. cisplatin; Figure 3, C and J), and *KIM1* (A: –95.2% ± 0.7%; SC: –79.5% ± 3.8% vs. cisplatin; Figure 3, D and K). KW6002 treatment also significantly reduced renal inflammation, as exemplified by the expression of *Tnfa* and *Il6* (Figure 3, E, F, L, and M) and other proinflammatory markers (Supplemental Table 1) as well as reduced apoptosis (Figure 3, G and N). Moreover, KW6002 also alleviated cisplatin-induced nephrotoxicity, as evidenced by reduced BUN levels as well as *NGAL* and *KIM1* expression levels (Supplemental Figure 2), in a cumulative model of cisplatin toxicity (Supplemental Figure 1E).

### Transcriptomic signature is associated with the protective effect of KW6002 on cisplatin-induced renal injury.

**Figure 4:**
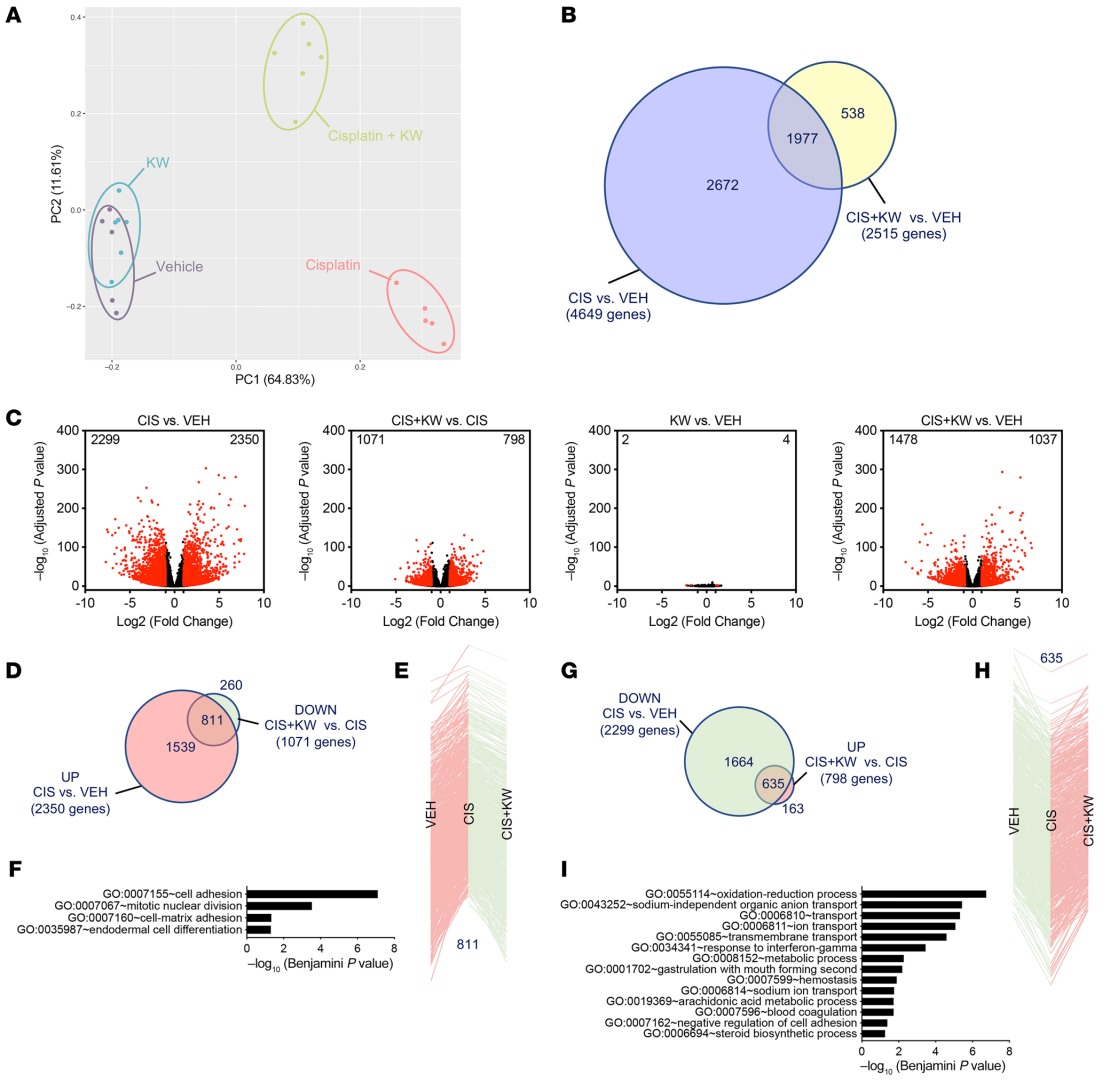
Transcriptomic analysis detailing the protective effect of KW6002 on renal toxicity induced by subchronic cisplatin administration. (**A**) PCA of kidney RNA-Seq (*n* = 5–6 per group). (**B**) Venn diagram representing the number of genes deregulated by cisplatin (CIS) alone (4,649 genes) or by KW6002 plus cisplatin (2,515 genes). (**C**) Volcano plots showing the number of renal genes significantly up- or downregulated in the different conditions. (**D**–**F**) Analysis of the 811 genes normalized by KW6002 coadministration among the 2,350 genes upregulated by cisplatin. (**D**) Venn diagram showing that a marked number of renal genes (*n* = 811) downregulated (DOWN) by KW6002 in cisplatin-treated animals overlapped with genes upregulated (UP) by cisplatin alone. (**E**) These 811 genes are represented according to their changes in expression due to treatment with cisplatin alone or with cisplatin plus KW6002. (**F**) GO enrichment analysis of the 811 genes both overexpressed following cisplatin treatment and repressed by KW6002 treatment. (**G**–**I**) Analysis of the 635 genes normalized by KW6002 coadministration among the 2,299 genes downregulated by cisplatin. (**G**) Venn diagram shows that a substantial number of renal genes (*n* = 635) upregulated by KW6002 in cisplatin-treated animals overlapped with genes downregulated by cisplatin alone. (**H**) These 635 genes are represented according to their changes in expression due to treatment with cisplatin alone or with cisplatin plus KW6002. (**I**) GO enrichment analysis of the 635 genes both overexpressed following cisplatin treatment and repressed by KW6002 treatment. VEH, vehicle.

To gain insights into the in vivo molecular events underlying the beneficial effects of A_2A_R antagonism in the injured kidney, we used an RNA-Seq transcriptomic approach in the subchronic protocol. Principal component analysis (PCA) of the experimental groups (*n* = 5–6 per group) is shown in Figure 4A. Cisplatin had a profound impact on the kidney transcriptome, affecting the expression of 4,649 genes (adjusted *P* < 0.01, log_2_ fold change ± 1), with 2,350 of these genes being upregulated and 2,299 downregulated (Figure 4, B and C). Interestingly, KW6002 reduced by approximately 50% the transcriptomic changes induced by cisplatin (Figure 4, B and C).

Among the 2,350 genes upregulated by cisplatin, 811 (~34%) were normalized by KW6002 coadministration (Figure 4, D and E), whereas KW6002 had almost no effect on the control mice (Figure 4C). Functional enrichment analyses performed using DAVID (Database for Annotation, Visualization, and Integrated Discovery) (https://david.ncifcrf.gov) showed that these 811 genes were notably associated with cell adhesion and proliferation (Figure 4F). Unsupervised gene set enrichment analysis (GSEA) of pathways downregulated by KW6002 in the kidney of cisplatin-treated animals further indicated that A_2A_R antagonism reduced kidney apoptosis (normalized enrichment score [NES] < –5; FDR < 1.73 × 10^–5^; Supplemental Table 2).

Among the 2,299 genes downregulated by cisplatin, 635 (~27%) were normalized by KW6002 cotreatment (Figure 4, G and H), and these genes were mainly associated with redox balance and transport processes (Figure 4I). Unsupervised GSEA analysis of pathways upregulated by KW6002 in the kidneys of cisplatin-treated animals confirmed the strong impact of A_2A_R antagonism on redox balance and metabolic processes (NES >5; FDR < 1.25 × 10^–5^; Supplemental Table 2). We additionally used Ingenuity Pathway Analysis (IPA) to predict molecular and cellular functions, toxicological features, or upstream regulators affected by cisplatin that were normalized by KW6002 cotreatment. Our analysis of these 635 KW6002-modulated genes identified networks and canonical pathways involved in kidney damage (Supplemental Figure 3, A and B and Supplemental Tables 3 and 4) and lipid metabolism (particularly fatty acid metabolism; Supplemental Figure 3C and Supplemental Tables 3 and 4), suggesting that the genes in these networks were specifically associated with the protective effects of KW6002 in cisplatin-treated kidneys. Finally, we performed upstream analysis of KW6002-modulated genes and identified several crucial upstream regulators such as hepatocyte nuclear factor 1 A isoform (HNF1A), LIM homeobox protein 1 (LHX1), and synuclein (SNCA), which are known to affect kidney functions and/or lipid homeostasis (Supplemental Table 5).

**Figure 5:**
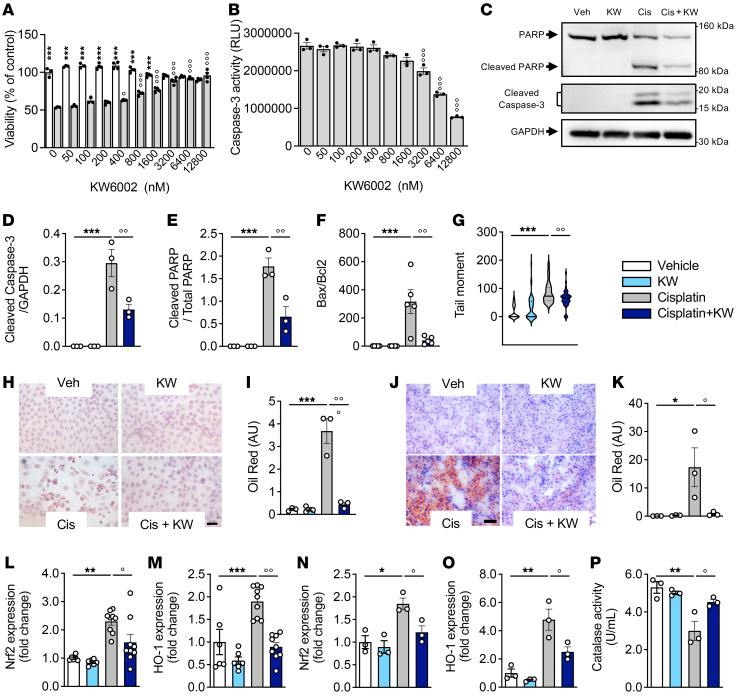
KW6002 alleviates the cisplatin-induced nephrotoxicity associated with apoptosis, lipid metabolism, and oxidative stress. (**A** and **B**) RPTEC/hTERT1 cells were coexposed to cisplatin (50 μM) and increasing concentrations of KW6002. Cell viability (**A**) and caspase 3/-7 activity (**B**) were determined 72 hours after treatment. ***P < 0.001 versus the respective cisplatin condition; °*P* < 0.01 and °°°*P* < 0.001 versus cisplatin, not treated with KW6002; 1-way ANOVA followed by Tukey’s post hoc test (*n* = 3–4/condition). (**C**–**I** and **N**–**P**) RPTEC/hTERT1 cells were exposed to cisplatin (50 μM) and/or KW6002 (25 μM). *n* = 3 independent experiments. (**C**–**E**) Representative Western blots (**C**) and quantification showing the cleavage of PARP and caspase 3 proteins (**D** and **E**). (**F**) *Bax/Bcl2* mRNA ratio. ***P < 0.001 versus vehicle; °°*P* < 0.01 versus cisplatin; 1-way ANOVA followed by Tukey’s post hoc test (*n* = 3 independent experiments). (**G**) Tail moment assessed by comet assay; 50 cells/condition were analyzed. (**H**–**K**) KW6002 attenuates cisplatin-induced renal lipid accumulation, assessed by Red Oil staining. Red color indicates lipid deposition. (**H** and **I**) Representative images of RPTEC/hTERT1 cells (scale bar: 20 μm) **(H)** and quantification (*n* = 5 randomly chosen fields/staining) (**I**). ***P < 0.001 versus vehicle; °°*P* < 0.01 versus cisplatin; 1-way ANOVA followed by Tukey’s post hoc test (*n* = 3 independent experiments). (**J** and **K**) Representative images of the subchronic cisplatin mouse model (scale bar: 200 μm) and (**K**) quantification (*n* = 8 randomly chosen fields/section) Results are the mean ± SEM. *P < 0.05 versus vehicle; °*P* < 0.05 versus cisplatin; 1-way ANOVA followed by Tukey’s post hoc test (*n* = 3 mice/group). (**L** and **M**) KW6002 attenuated cisplatin-induced oxidative stress in the subchronic cisplatin mouse model. Gene expression of *Nrf2* (**L**) and *HO1* (**M**). **P < 0.01 and ***P < 0.001 versus vehicle; °*P* < 0.05 and °°*P* < 0.01 versus cisplatin; 1-way ANOVA followed by Tukey’s post hoc test. Results are the mean ± SEM (*n* = 6–9 mice/group). (**N**–**P**) KW6002 attenuated cisplatin-induced oxidative stress in RPTEC/hTERT1 cells. (**N** and **O**) Gene expression of *Nrf2* (**N**) and *HO1* (**O**). (**P**) Catalase activity. *P < 0.05 and **P < 0.01 versus vehicle; °*P* < 0.05 versus cisplatin; 1-way ANOVA followed by Tukey’s post hoc test. Results are the mean ± SEM (*n* = 3 independent experiments).

The above-mentioned KW6002-regulated pathways uncovered by RNA-Seq analysis were confirmed by additional in vitro and in vivo analyses. First, using a human proximal tubular epithelial cell line (RPTEC/hTERT1), we found that KW6002 concentration-dependently reduced cisplatin-induced cell death (Figure 5A), in particular by inhibiting cisplatin-induced apoptosis (Figure 5, B–F) as well as DNA damage (Figure 5G). Furthermore, cisplatin-induced lipid accumulation was significantly reduced by KW6002 in both RPTEC/hTERT1 cells (Figure 5, H and I) and kidney samples (Figure 5, J and K). Finally, KW6002 normalized the cisplatin-induced increase in the expression of 2 master regulators of oxidative stress — *Nrf2* and *HO1* — both in vivo (Figure 5, L and M) and in vitro (Figure 5, N and O) as well as the decrease in catalase activity (Figure 5P).

### Effect of KW6002 in tumor-bearing mice.

**Figure 6:**
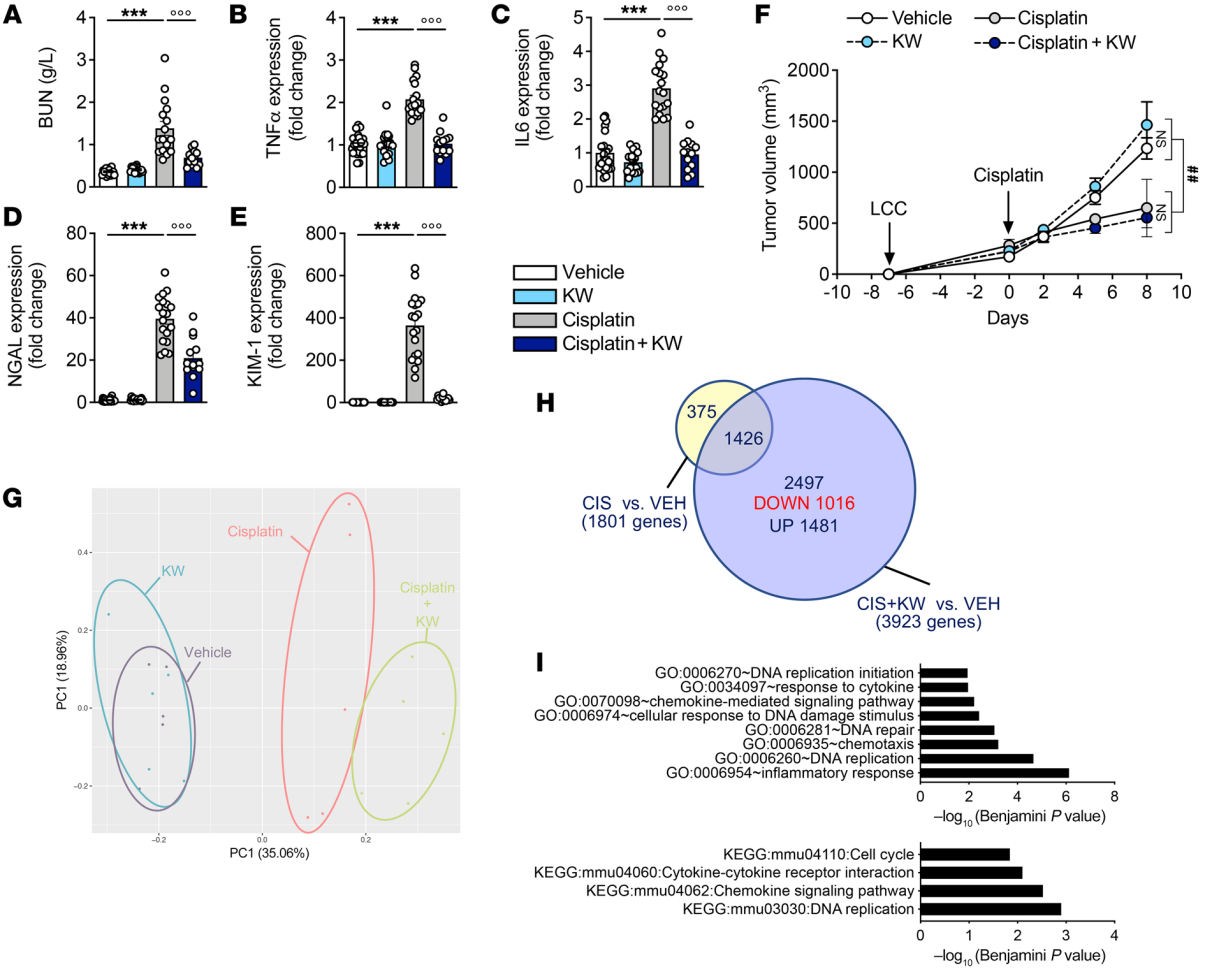
KW6002 protects against nephrotoxicity without attenuating the antitumoral properties of cisplatin in an LLC1 syngeneic in vivo mouse model. (**A**–**E**) KW6002 alleviates cisplatin-induced nephrotoxicity as estimated by BUN quantification (**A**), mRNA levels of inflammatory markers (*Tnfa* and *IL6*) (**B** and **C**), and renal injury markers (*NGAL* and *KIM1*) (**D** and **E**). (**F**) Absolute tumor sizes in the different groups of animals. Results are the mean ± SEM. ***P < 0.001 versus vehicle; °°°*P* < 0.001 versus cisplatin; 1-way ANOVA followed by Tukey’s post hoc test (*n* = 13–25 animals/group). ^##^*P* < 0.01; 2-way ANOVA (*n* = 6–10 animals per group). (**G**) PCA from tumor RNA-Seq (*n* = 5 per group). (**H**) Venn diagram representing the number of tumor genes dysregulated by cisplatin or KW6002 plus cisplatin. (**I**) GO enrichment analysis and KEGG (Kyoto Encyclopedia of Genes and Genomes) pathway enrichment of the 1,016 tumor genes downregulated by KW6002 in the cisplatin-treated condition.

From a clinical perspective, it was crucial to determine whether KW6002 preserved kidney function after cisplatin exposure without, at minima, compromising its antitumoral efficacy. To address this question, we first used the syngeneic LLC1 lung cancer mouse model (39). Subcutaneous LLC1 tumors were induced in C57BL6/J mice, which were then treated with cisplatin, KW6002, or both (Supplemental Figure 1F). In this model, we could then simultaneously gauge whether KW6002 modulated the effects of cisplatin toward kidney injury and tumorigenicity. We observed that the ability of KW6002 to protect from cisplatin-induced kidney injury was preserved in tumor-bearing mice (Figure 6, A–E). Importantly, KW6002 did not compromise the antitumoral response to cisplatin in this model (Figure 6F). Moreover, we conducted a whole-transcriptome analysis to decipher potential molecular changes occurring in tumors in response to cisplatin and/or KW6002. The PCA (*n* = 5 per group) results are shown in Figure 6G. Compared with vehicle-treated mice, KW6002 did not modulate gene expression in tumors (data not shown). Compared with vehicle-treated mice, cotreatment of mice with cisplatin and KW6002 changed the expression levels of 3,923 genes (adjusted *P* < 0.05, log_2_ fold change ± 0.32), while cisplatin alone altered the transcription of 1,801 genes. Therefore, the impact of cisplatin on the tumor cell transcriptome was enhanced in the presence of KW6002 (Figure 6H). Among the 2,497 (of 3,923) genes selectively modulated by cisplatin and KW6002 cotreatment versus vehicle, 1,016 genes were downregulated, and their annotation particularly referred to chemokine and cytokine responses, the cell cycle, as well as DNA repair and replication (Figure 6I and Supplemental Figure 4). Unsupervised GSEA analysis of pathways upregulated by KW6002 in LLC1 tumors of cisplatin-treated animals also suggested the effect of A_2A_R antagonism on DNA repair and replication (NES < –4; FDR < 3.04 × 10^–5^; Supplemental Table 6). Using IPA, we further identified the most significant diseases, molecular and cellular functions, and biological networks related to these 1,016 genes that were specifically downregulated by KW6002 upon cisplatin cotreatment. The altered gene expression patterns were particularly related to cancer (Supplemental Table 7), with biological gene networks linked, for example, to “cancer, hematological disease, immunological disease” or “cancer, cardiovascular disease, DNA replication, recombination, and repair” (Supplemental Table 8).

**Figure 7:**
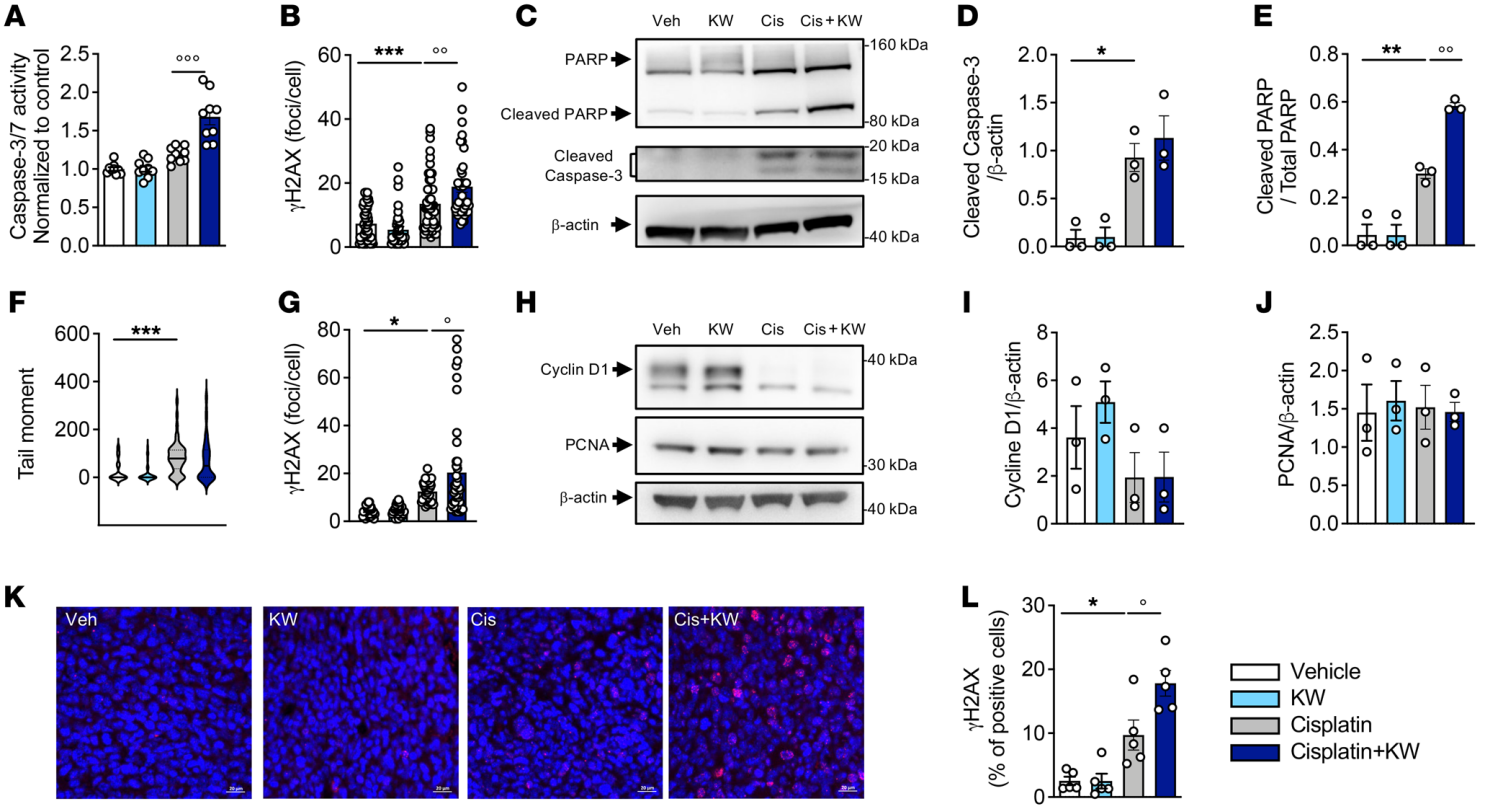
KW6002 does not interfere with the antitumoral effect of cisplatin. (**A** and **B**) LLC1 cells were exposed to 2 μM cisplatin with or without 10 nM KW6002 for 24 hours (**A**) or 6 hours (**B**). Caspase 3/-7 activity (*n* = 3 independent experiments) (**A**) and the number of γH2AX nuclear foci (*n* = 36–63 nuclei/group) (**B**) were determined. Results are the mean ± SEM. ***P < 0.001 versus vehicle; °°*P* < 0.01 and °°°*P* < 0.001 versus cisplatin; 1-way ANOVA followed by Tukey’s post hoc test. (**C**–**E**, **I**, and **J**) H1975 cells were exposed to 20 μM cisplatin with or without 10 nM KW6002 for 24 hours. Representative Western blot shows cleaved PARP and caspase 3 proteins (**C**) as well as cyclin D1 and PCNA (**H**) expression. Quantification of caspase 3 (**D**), PARP (**E**), cyclin D1 (**I**), and PCNA (**J**). *P < 0.05 and **P < 0.01 versus vehicle; °°*P* < 0.01 versus cisplatin; 1-way ANOVA followed by Tukey’s post hoc test. Results are expressed as the mean ± SEM (*n* = 3 independent experiments). (**F**) Tail moment was assessed by comet assay. A total of 50 cells per condition were analyzed. ***P < 0.001; 1-way ANOVA followed by Tukey’s post hoc test. (**G**) H1975 cells were exposed to 50 μM cisplatin with or without 10 nM KW6002 for 6 hours, and the number of γH2AX nuclear foci was counted. Results are expressed as the mean ± SEM. *P < 0.05 versus vehicle; °*P* < 0.01 versus cisplatin; 1-way ANOVA followed by Tukey’s post hoc test (*n* = 30–42 nuclei/condition). (**K** and **L**) KW6002 increased γH2AX expression in tumors from mice of the LLC1 syngeneic model. (**K**) Representative images of nuclear γH2AX staining (red) in the different experimental conditions. Scale bars: 20 μm. (**L**) Quantification of nuclear γH2AX expression. The percentage of positive cells was calculated using 5 randomly chosen fields per staining. *P < 0.05 versus vehicle; °*P* < 0.01 versus cisplatin; 1-way ANOVA followed by Tukey’s post hoc test (*n* = 5/group).

In agreement with this pathway analysis, in vitro studies performed using 2 cancerous cell lines (LLC1 and H1975) confirmed that KW6002 did not impede the antitumoral effect of cisplatin in terms of apoptosis (Figure 7, A and C–E), DNA damage (Figure 7, B, F, and G), and the cell cycle (Figure 7, H–J). Of note, KW6002 even potentiated the efficacy of cisplatin (Figure 7, A, B, E, G, J–L), with a particular effect on cisplatin-induced DNA damage, as evidenced by the increased number of H2AX^+^ cells observed both in vitro in LLC1 and H1975 cells (Figure 7, B and G) as well as in vivo in the LLC1 syngeneic model (Figure 7, K and L).

### KW6002 limits renal accumulation of cisplatin.

**Figure 8:**
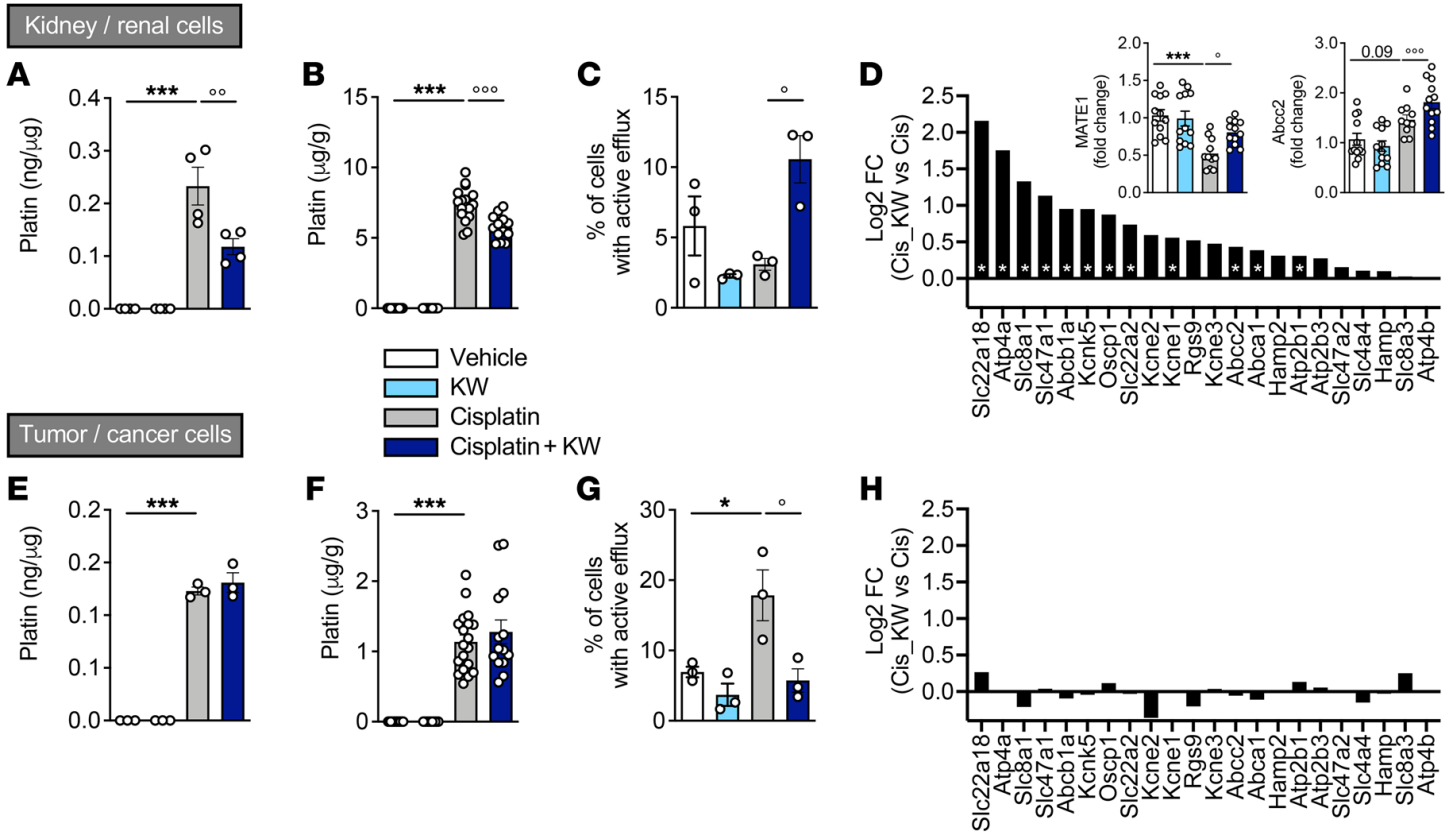
KW6002 limits cisplatin accumulation in the kidney but not in tumors. RPTEC/hTERT1 and H1975 cells were exposed to cisplatin and/or KW6002. *n* = 3 independent experiments. (**A** and **E**) Platinum quantification (ng/μg proteins) in RPTEC/hTERT1 (**A**) and H1975 (**E**) cells. ***P < 0.001 versus vehicle; °°*P* < 0.01 versus cisplatin; 1-way ANOVA followed by Tukey’s post hoc test. Results are the mean ± SEM (*n* = 3–4 independent experiments). (**B** and **F**) Platinum quantification in kidney (**B**) and tumor (**F**) samples from mice of the LLC1 syngeneic model. ***P < 0.001 versus vehicle; °°°*P* < 0.001 versus cisplatin; 1-way ANOVA followed by Tukey’s post hoc test. Results are the mean ± SEM (*n* = 12–20/group). (**C** and **G**) Active efflux in RPTEC/hTERT (**C**) and H1975 (**G**) cells. *P < 0.05 versus vehicle; °*P* < 0.05 versus cisplatin; 1-way ANOVA followed by Tukey’s post hoc test. Results are the mean ± SEM (*n* = 3 independent experiments). (**D** and **H**) Relative expression from RNA-Seq of 22 genes annotated as “export across plasma membrane” (extracted from G0: 0055085 transmembrane transport) in kidney (**D**) and tumor (**H**) samples from mice of the LLC1 syngeneic model (*n* = 5–6/group). Inserts represent the relative expression of *Mate1* and *Abcc2* evaluated by quantitative PCR in kidney samples. Results are the mean ± SEM. *P < 0.05 and ***P < 0.001 versus vehicle; °*P* < 0.05 and °°°*P* < 0.001 versus cisplatin; 1-way ANOVA followed by Tukey’s post hoc test (*n* = 10–12/group).

To understand how KW6002 alleviates cisplatin-induced nephrotoxicity while preserving the ability of cisplatin to control tumor growth, we quantified platinum levels both in vitro and in vivo. Platin accumulated in cisplatin-treated renal RPTEC/hTERT1 and cancer H1975 cells (Figure 8, A and E). KW6002 significantly reduced platin accumulation in RPTEC/hTERT1 cells but not in H1975 cells (Figure 8, A and E). Consistently, in KW6002-treated mice, we found that cisplatin accumulation was lowered in the kidney, while it remained unchanged in the tumors (Figure 8, B and F). This observed discrepancy between renal and cancer cells led us to evaluate the efflux ability of renal RPTEC/hTERT1 and cancer H1975 cells by flow cytometry. While efflux was significantly reduced by KW6002 in cancer cells in response to cisplatin (Figure 8G), it was strongly enhanced by KW6002 in cisplatin-treated RPTEC/hTERT1 cells (Figure 8C). Such a differential effect of KW6002 on cisplatin accumulation and efflux in kidneys and tumors might be explained by different expression profiles of genes involved in export across the plasma membrane. Indeed, RNA-Seq experiments indicated that the expression of several efflux transporters remained unchanged in tumors (Figure 8H), while it was significantly increased by KW6002 in kidney (Figure 8D), including the transporters multidrug and toxin extrusion 1 (MATE1, also known as Slc47a1) and Abcc2, whose modulation was validated by quantitative PCR (insets in Figure 8D).

### A_2A_R antagonism limits cisplatin-induced pain hypersensitivity.

**Figure 9:**
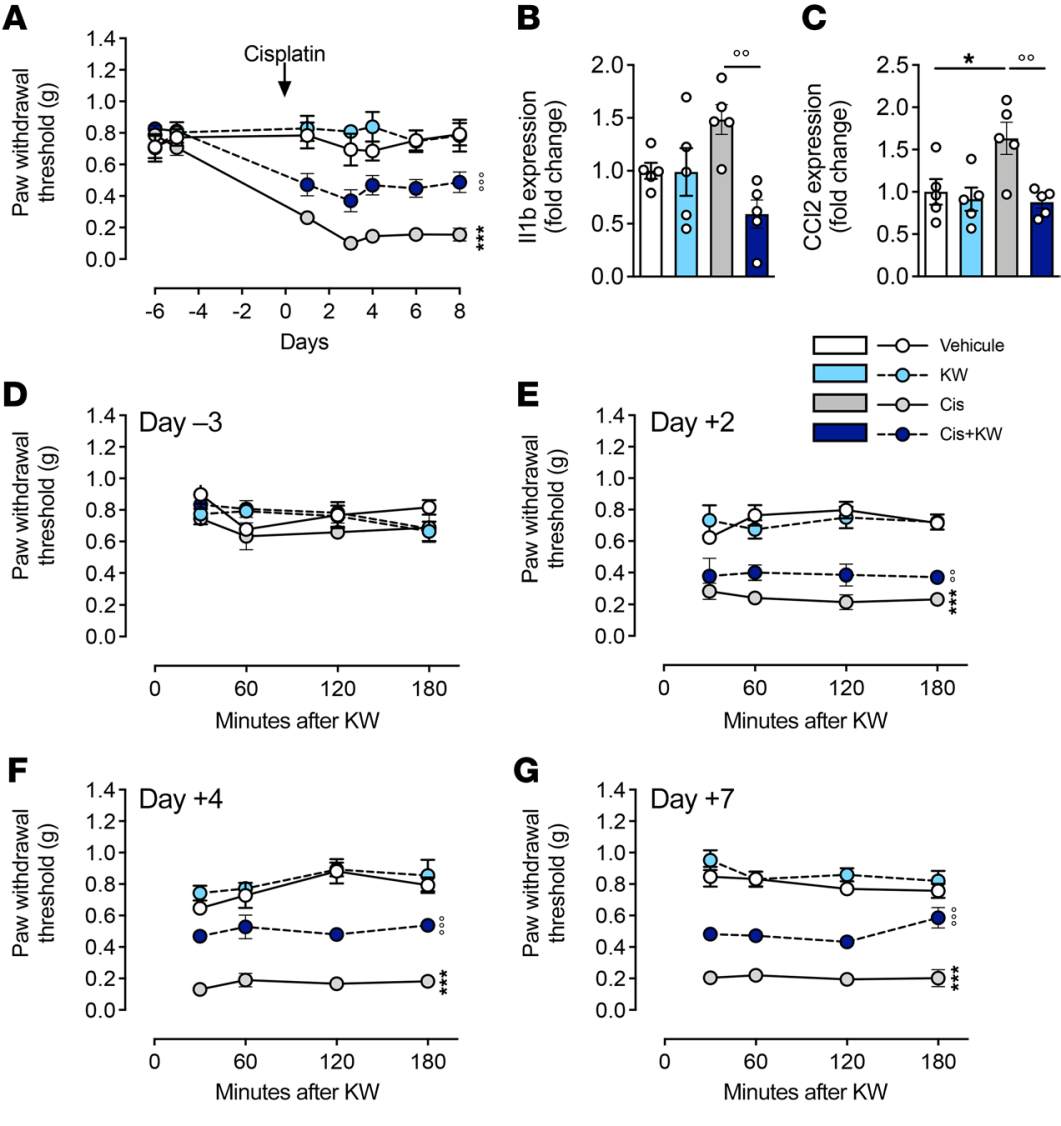
KW6002 reduces the pain hypersensitivity and cytokine upregulation induced by cisplatin. (**A**) Mechanical sensitivity measured by von Frey hairs in mice treated with cisplatin and/or KW6002 in the subchronic model described in Supplemental Figure 1D. The arrow represents cisplatin and/or PBS injection. Pain was measured 24 hours after KW6002 or vehicle injection. Data are the mean ± SEM. ***P < 0.001 versus vehicle; °°°*P* < 0.001 versus cisplatin; 2-way ANOVA (*n* = 5/group). (**B** and **C**) mRNA levels of *Il1b* (**B**) and *Ccl2* (**C**) in DRGs 8 days after the start of cisplatin treatment. Data are the mean ± SEM. *P < 0.05 versus vehicle; °°*P* < 0.01 versus cisplatin; 2-way ANOVA (*n* = 5/group). (**D**–**G**) Time-course evaluation of mechanical sensitivity in mice measured by von Frey hairs during the first 180 minutes following KW6002 administration in response to cisplatin on day –3 (D–3) (**D**), day +2 (D+2) (**E**), day +4 (**F**), and day +7 (**G**). Data are the mean ± SEM. °*P* < 0.05 versus cisplatin; 1-way ANOVA followed by Tukey’s post hoc test (*n* = 5/group).

Another important limitation in the therapeutic use of cisplatin is the occurrence of CIPN, in particular, pain hypersensitivity (40, 41). To evaluate whether KW6002 also alleviates cisplatin-induced pain hypersensitivity and the associated burst of proinflammatory cytokines in the dorsal root ganglion (DRG) and spinal cord, we treated mice with cisplatin and KW6002 as described above (Supplemental Figure 1D). We found that treatment with KW6002 significantly mitigated pain hypersensitivity (Figure 9A) and reversed the upregulation in the DRG of *Il1b* and *Ccl2* (Figure 9, B and C), 2 cytokines known to contribute to CIPN (42, 43). We observed that other inflammatory mediators, upregulated by cisplatin and decreased by KW6002, overlapped between the DRG and kidney (Supplemental Table 1). To further understand the effect of KW6002 on cisplatin-induced pain hypersensitivity, we performed, at different time points of cisplatin intoxication, a time-course evaluation of pain sensitivity following KW6002 injection. We found that KW6002 did not show an acute analgesic effect but rather a cumulative and persistent effect (Figure 9, D–G). These data support the idea that, in addition to alleviating nephrotoxicity, KW6002 can also mitigate cisplatin-induced peripheral neuropathy.

### A_2A_R antagonism protects against cisplatin-induced nephrotoxicity and CIPN, while enhancing tumor growth control in a syngeneic model of HPV^+^ squamous carcinoma.

**Figure 10:**
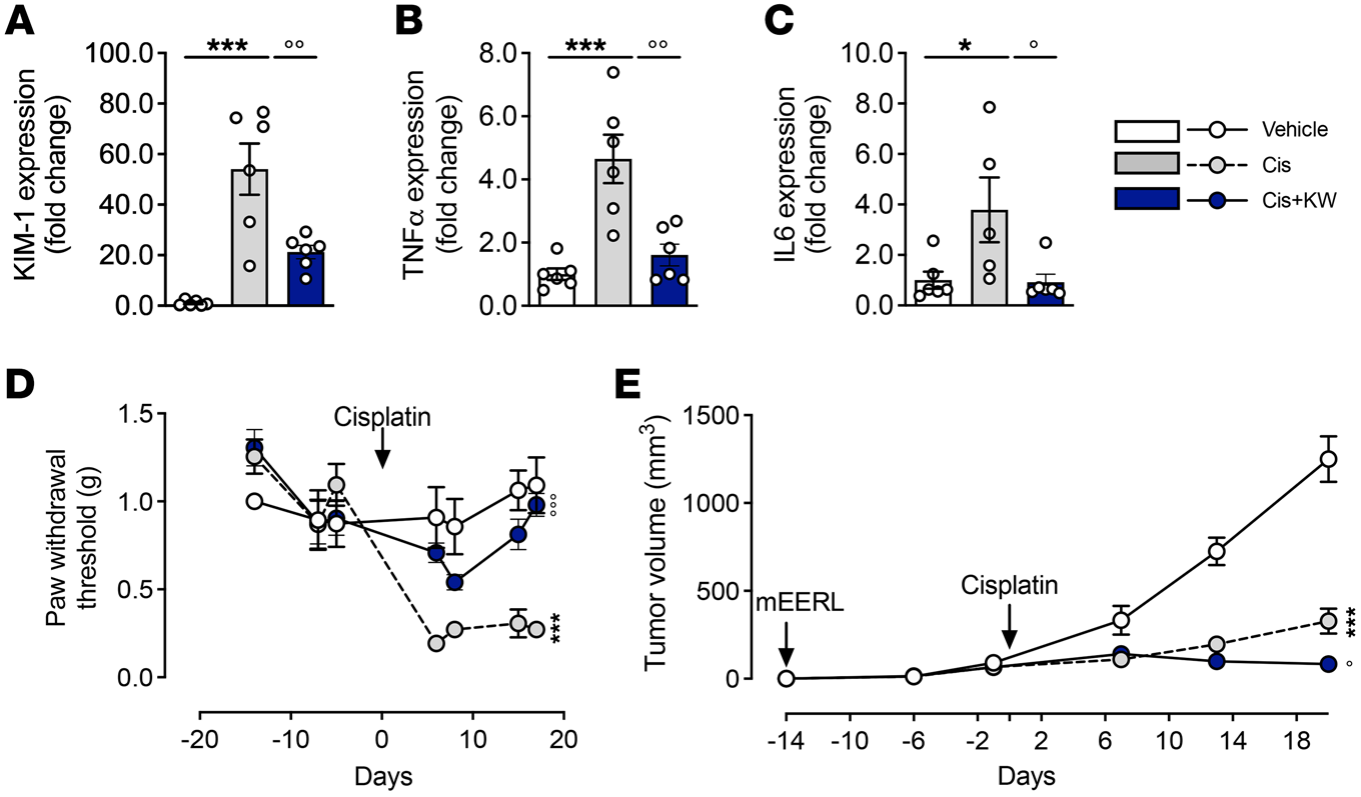
KW6002 prevents nephrotoxicity and neurotoxicity without attenuating the antitumoral properties of cisplatin in the mEERL syngeneic in vivo mouse model. (**A**–**C**) KW6002 alleviated cisplatin-induced nephrotoxicity as estimated by mRNA levels of *KIM1* (**A**) and the inflammatory markers *Tnfa* and *Il6* (**B** and **C**). *P < 0.05 and ***P < 0.001 versus vehicle; °*P* < 0.01 and °°*P* < 0.01 versus cisplatin; 1-way ANOVA (*n* = 5–6/group). (**D**) Mechanical sensitivity measured by von Frey hairs in mice in response to cisplatin and/or KW6002. The arrow represents cisplatin and/or PBS injection. Data are the mean ± SEM. ***P < 0.001 versus vehicle; °°°*P* < 0.001 versus cisplatin; 2-way ANOVA (*n* = 5/group). (**F**) Absolute tumor sizes in animals in the different groups. Results indicate the mean ± SEM. ***P < 0.001 versus vehicle; °*P* < 0.05 versus cisplatin; 2-way ANOVA (*n* = 5–6 animals/group).

We validated the nephro- and neuroprotective effects of KW6002 in a tumoral context using an additional cancer mouse model (44). Subcutaneous mEERL cells were injected into C57Bl6/J mice, which were then treated with cisplatin alone or in combination with KW6002, as indicated in Supplemental Figure 1G. KW6002 administration in tumor-bearing mice limited indicators of renal toxicity (KIM-1, Figure 10A) and expression of the inflammatory cytokines *Tnf* and *Il6* (Figure 10, B and C) induced by cisplatin. KW6002 also alleviated cisplatin-induced pain hypersensitivity in this model (Figure 10D). Finally, KW6002 significantly potentiated tumor control by cisplatin (Figure 10E). Overall, using this additional model with a different cancer etiology, the nephroprotective effect, the reduction of pain hypersensitivity, and the potentiation of tumor control were replicated, highlighting the promising therapeutic potential of A_2A_R inhibition.

## Discussion

Cisplatin-induced nephrotoxicity and peripheral neuropathy remain serious adverse effects, affecting approximately one-third of exposed patients (12, 13). Identifying targets to alleviate such toxicities without lessening tumor control by cisplatin is therefore a major clinical challenge. Moreover, an optimal therapeutic solution would ideally act synergistically with cisplatin to promote cancer regression, while protecting kidney and sensory functions. In the present study, we provided evidence that administration of the A_2A_R antagonist istradefylline (KW6002) efficiently and reproducibly prevented the nephrotoxicity and pain hypersensitivity that are induced by single or repeated administration of cisplatin in mice. These beneficial effects were observed while the tumor growth control properties of cisplatin were preserved.

Our targeted and nontargeted (RNA-Seq) experiments indicated that cisplatin affects renal function by promoting cell death via multiple pathways including those for the inflammatory response, redox balance, intracellular lipid accumulation, transport impairment and apoptotic induction (45–47). Treatment with KW6002 probably alleviated the latter, as shown in vivo but also in vitro using the renal proximal tubule epithelial cell/hTERT1 (RPTEC/hTERT1) cell line. The effects of KW6002 on lipids and oxidation are of particular interest. Indeed, fatty acid metabolism represents an essential energy resource for the production of ATP in renal tubules (48, 49). According to recent reports, impaired fatty acid oxidation, which ultimately causes lipid accumulation and PTEC injury, plays a key role in the process of cisplatin nephrotoxicity (49, 50). Cisplatin is highly reactive toward nucleophilic substances such as glutathione (GSH), cysteines, or methionines, which are metabolically activated to form reactive thiols (3, 51). Accumulation of cisplatin in the mitochondria of tubular epithelial cells then increases the levels of ROS and decreases the levels of antioxidant components such as catalase, GSH, and superoxide dismutase (45, 52), leading to oxidative stress–related damage and death of proximal tubule epithelial cells (53, 54). The effect of KW6002 on the renal redox balance is therefore of particular importance and in line with previous studies showing that A_2A_R antagonists can counteract oxidative stress in different cell types and tissues (55, 56).

Our data also suggest that the nephroprotective effect of KW6002 additionally relied on its ability to limit platinum accumulation in the kidney. Platinum accumulation is consistently associated with the upregulation of ABCC2 and MATE-1, the main transporters previously identified to be involved in cisplatin efflux (8). In line with this, upregulation of MATE-1 has been shown to increase the efflux of cisplatin from renal cells (57), while genetic deletion of MATE-1 exacerbates cisplatin nephrotoxicity in mice (58). How KW6002 regulates efflux transporter expression remains unclear; however, A_2A_R activation was previously reported to decrease the expression and function of P-glycoprotein (also known as ABCB1), a member of the same family as ABCC2, leading to the accumulation of P-glycoprotein substrates in the mouse brain (59). The localization of A_2A_R in kidney-resident cells and especially in epithelial tubular cells of cisplatin-treated animals and the fact that KW6002 limits the accumulation of cisplatin in the RPTEC/hTERT1 proximal tubular epithelial cell line in vitro are in favor of a direct effect of KW6002 on the A_2A_R on tubular cells. Similarly, the local production of TNF-α by renal parenchymal cells is likely contributing to cisplatin-induced nephrotoxicity (60). However, we cannot rule out the possibility that KW6002 might exert its beneficial effect by modulating A_2A_Rs located on inflammatory cells, as supported by the reduced expression of *Tnf* and *Il6*. Extracellular adenosine has been indeed shown to be important for the regulation of immune cell activation in the kidney, in particular in the context of renal ischemia; however, activation rather than blockade of A_2A_R signaling is acknowledged for its immunosuppressive effect (31, 32, 61).

In addition to its nephroprotective effects, KW6002 also alleviated cisplatin-induced pain hypersensitivity, a common sign of CIPN (41), by reducing the expression of proinflammatory and proalgesic cytokines in the DRG. Whether the mechanisms underlying KW6002 actions in the DRG are similar to those in the kidney will be the focus of future studies. Further studies should also clarify if A_2A_R antagonists might also limit neuropathy-related side effects of other chemotherapeutic agents with different modes of action.

It is clinically highly relevant that KW6002 exerts potent effects on cisplatin-induced renal toxicity, without affecting cisplatin’s antitumoral properties. Indeed, the reduced tumor growth rate induced by cisplatin was not affected by KW6002 cotreatment in a LLC1 syngeneic model and was even enhanced in a mEERL syngeneic model. Adenosine levels are particularly elevated in the tumor microenvironment (62, 63), impairing antitumor immunity, notably through the activation of the A_2A_R present in immune cells (14, 64). Accordingly, A_2A_R antagonists are currently being explored in clinical trials as coadjuvants for autoimmune transplantation therapies for immunogenic cancers (NCT05024097, https://clinicaltrials.gov/ct2/show/NCT05024097?term=adenosine+receptor&cond=cancer&draw=2&rank=1). Interestingly, platinum-based chemotherapeutic agents have been suggested to promote an adenosine surge by cancer cells, conferring chemoresistance and further suppressing antitumor immunity (64). In this context, A_2A_ receptor blockade is currently seen as a valuable strategy to improve chemotherapy through immune-oncological effects (64, 65). Besides the impact of KW6002 on mEERL tumor control in vivo, the molecular analysis of syngeneic LLC1 tumors from animals treated with cisplatin demonstrated that cotreatment with KW6002 led to a major reduction of molecular pathways related to cancer, notably cell growth pathways, such as those for DNA replication and repair. Interestingly, IPA analysis from in vivo tumors also predicted necrosis and apoptosis to be particularly activated in tumors (*P* = 2.78 × 10^−22^), in agreement with our in vitro experiments supporting a synergic effect of cisplatin and KW6002 on both mouse (LLC1) and human (H1975) cancer cells. Moreover, in response to cisplatin, efflux was markedly reduced by KW6002 in cancer cells, thus preserving the intracellular cisplatin concentration. Taken together, our data suggest that KW6002 bolstered the antitumoral properties of cisplatin through a combined A_2A_R-mediated increase in the susceptibility of cancer cells, together with antitumor immunity. This contention is consistent with previous data highlighting that caffeine, a nonselective adenosine receptor antagonist, potentiates the antitumoral effect of cisplatin both in vitro and in vivo (66, 67).

In conclusion, our study clearly demonstrates the high efficiency of KW6002 in attenuating cisplatin-induced nephrotoxicity and peripheral neuropathy without compromising the antitumoral properties of cisplatin. Considering the safety and tolerability of the FDA-approved KW6002 (36), our data prompt its clinical repurposing in patients with cancer undergoing cisplatin chemotherapy. In addition, we describe here a molecular target that efficiently circumvented 2 major cisplatin side effects, strengthening the potential clinical value of A_2A_R pharmacological modulation.

## Methods

### Animals and treatments.

Animal experiments were adapted from previous work (68, 69). Animal procedures were performed in 8- to 10-week-old male C57Bl6/J mice (Janvier Labs except for mice used in the pain experiments, which were obtained from The Jackson Laboratory). Mice were fed a laboratory standard diet with water and food ad libitum and were kept under constant environmental conditions with a 12-hour light/12-hour dark cycle. Istradefylline (KW6002, Tocris) was dissolved in a carrier solution consisting of 15% DMSO, 15% cremophor (MilliporeSigma), and 70% saline solution (vehicle). Cisplatin (Accord Healthcare) was dissolved in saline solution. Acute cisplatin nephrotoxicity was induced following a single i.p. injection (day 0) of 10 mg/kg cisplatin. Three days after this single injection, animals were sacrificed by cervical dislocation (Supplemental Figure 1A). When KW6002 (3 mg/kg) was tested against acute cisplatin toxicity, the drug was administered daily i.p. from day –1 to day 2 (Supplemental Figure 1C). Toxicity of subchronic cisplatin was evaluated following 6 daily i.p. injections of cisplatin (3 mg/kg) starting on day 0, and mice were sacrificed 72 hours after the last injection of cisplatin (day 8; Supplemental Figure 1B). When KW6002 was tested against subchronic cisplatin toxicity, the drug was administered i.p. daily from day –5 to day 7 (Supplemental Figure 1D). When KW6002 was tested against cumulative toxicity of cisplatin, KW6002 was administrated i.p. daily from day –5 to day 28. Mice were given daily i.p. injections of cisplatin (2.3 mg/kg; day 0) for 5 days, followed by 5 days of rest before a new cycle of 5 days of i.p. injections of cisplatin (2.3 mg/kg; day 10). Mice were sacrificed 5, 9, 15, or 28 days after the first cisplatin injection (Supplemental Figure 1E).

### LLC1 in vivo tumor model.

Lewis lung cancer (LLC1) cells (CRL-1642, American Type Culture Collectin [ATCC]) were cultured in DMEM with 10% FCS and penicillin-streptomycin. LLC1 cells (10^6^ cells) in PBS/Matrigel (1:1, for a total volume of 100 μL) were injected s.c. into the right flank of the animals. Tumors were measured twice a week with calipers, and tumor volumes were estimated using the following equation: ½ (length × width^2^). When tumor volume reached 100 mm^3^, mice were randomly ascribed to1 of the 4 experimental groups (vehicle; KW6002; cisplatin; or cisplatin plus KW6002), as indicated in Supplemental Figure 1E.

### mEERL in vivo tumor model.

We used a validated murine model of HPV^+^ oropharyngeal squamous cell carcinoma as previously described (noncommercial) (44) (Supplemental Figure 1G). This model consists of oropharyngeal epithelial cells from C57Bl/6 male mice that stably express the HPV16 viral oncogenes E6 and E7, H-Ras, and luciferase (mEERL cells). mEERL cells were grown in a T75 flask until confluent, after which cells were trypsinized and harvested, washed 3 times with sterile PBS, and resuspended in 1 mL sterile PBS to the appropriate concentration. Mice were injected s.c. into the right flank with 20 μL solution containing either 1,000,000 mEERL cells or PBS (vehicle). The day of mEERL cell injection is indicated as day –14. Tumor volume was monitored using Vernier digital calipers. When the tumor volume reached 100 mm^3^, the mice were randomly ascribed to 1 of the 3 experimental groups (vehicle; cisplatin; or cisplatin plus KW6002).

### Behavioral assessment.

Mechanical pain sensitivity was assessed using von Frey filaments as previously described (70, 71). Briefly, mice were placed in transparent boxes (10 × 10 × 10 cm). After a 30-minute habituation period, von Frey filaments were applied, and the paw withdrawal threshold was calculated using the “up and down” method. Behavioral testing was performed by experimenters blinded to the treatments.

### Sample collection.

Prior to sacrifice by cervical dislocation, retro-orbital blood samples were collected in heparinized tubes and centrifuged for 10 minutes at 900*g* at room temperature. Renal function was assessed by BUN measurement using a AU480 Chemistry Analyzer (Beckman Coulter). At the time of sacrifice, kidneys or LLC1 tumors were harvested and stored in either “RNA later” solution (Thermo Fisher Scientific) or 4% neutral buffered formalin or snap-frozen in liquid nitrogen. Lumbar DRG and spinal cord tissues were quickly dissected and snap-frozen in liquid nitrogen.

### Renal histological analysis.

Formalin-fixed, paraffin-embedded sections (3 μm thick) were stained with H&E (MilliporeSigma) or periodic acid-Schiff (MilliporeSigma). Slices were scored by a nephropathologist in a blinded manner. A kidney injury score grading scale from 0 to 5 was used to assess the severity of the injury as follow: 0 = no lesions; 1 = minimal injury characterized by the occurrence of necrosis and debris; 2 = mild injury with single-cell necrosis, pyknotic cells, and apoptosis; 3 = moderate injury characterized by tubular distension, vacuolation, and some cellular debris; 4 = severe injury with occasional hyaline casts observed, patchy epithelial necrosis in all segments, and loss of epithelial lining; and 5 = very severe injury characterized by extensive tubular epithelial necrosis in all segments, loss of the epithelial layer from many tubules, widespread intraluminal cellular debris, and frequent hyaline casts particularly prominent in the medullary region (72).

### Cell cultures.

RPTECs immortalized with a pLXSN-hTERT1 retroviral vector (CRL-4031, ATCC) are a relevant in vitro model to evaluate cisplatin’s deleterious effects (73–75). Cells were cultured in DMEM with F12 medium (DMEM and Ham’s F12 Medium, Thermo Fisher Scientific) supplemented with 1% penicillin-streptomycin, 5 pmol/L triiodo-l-thyronine, 10 ng/mL recombinant human EGF, 3.5 μg/mL ascorbic acid, 5.0 μg/mL human transferrin, 5.0 μg/mL insulin, 25 ng/mL prostaglandin E1, 25 ng/mL hydrocortisone, 8.65 ng/mL sodium selenite, 0.1 mg/mL G418, and 1.2 g/L sodium bicarbonate (MilliporeSigma). Murine LLC1 cells were cultured in DMEM GlutaMAX (Thermo Fisher Scientific) containing 10% FCS and 1% penicillin-streptomycin. Human lung adenocarcinoma NCI-H1975 cells (ATCC) were cultured in RPMI GlutaMAX (Thermo Fisher Scientific) containing 10% FCS and 1% penicillin-streptomycin. Cells were cultured at 37°C in a humidified atmosphere of 5% CO_2_.

### Cell viability assay.

RPTEC/hTERT1 cells were cultured in 96-well plates (40,000 cells/well) and exposed to cisplatin (50 μM) with or without KW6002 (0.5–12.8 μM) for 48 hours. Viability was assessed using the CellTiter-Glo Luminescent Cell Viability Assay (Promega) according to the manufacturer’s recommendations.

### Caspase 3/-7 activity.

RPTEC/hTERT1 cells were cultured in 96-well plates (40,000 cells/well) and exposed to cisplatin (50 μM) with or without KW6002 (25 μM) for 48 hours. LLC1 cells were cultured in 96-well plates (10,000 cells/well), and after 24 hours, the cells were exposed for 24 hours to 2 μM cisplatin with or without 10 nM KW6002. Apoptosis was assessed in RPTEC/hTERT1 and LLC1 cell lysates and in renal tissues using the Caspase-Glo 3/7 assay (Promega) according to the manufacturer’s recommendations.

### Catalase activity.

RPTEC/hTERT1 cells were cultured in 6-well plates (250,000 cells/well) and exposed for 48 hours to cisplatin (50 μM) with or without KW6002 (25 μM). Catalase activity was assessed using the Catalase Colorimetric Activity Kit (Thermo Fisher Scientific) according to the manufacturer’s instructions.

### Cell efflux.

The basic cell efflux function was assessed using an EFLUXX-ID Green Multidrug Resistance Assay Kit (ENZO Life Sciences). Briefly, 2.5 × 10^5^ cells/condition were collected, washed with PBS, and incubated with the EFLUXX-ID Green Detection Reagent for 30 minutes at 37°C, and then efflux was measured immediately by flow cytometry (CytoFLEX LX, Beckman Coulter). All experiments were performed in triplicate, with the measurement of 10,000 individual cells. Data were analyzed using Kaluza Analysis Software (Beckman Coulter).

### Comet assay.

Treated cells were suspended (60,000 cells/mL) in low-melt agarose (1613111, Bio-Rad) 0.5% in PBS at 42°C. The suspension was then immediately spread on a comet slide (4250-200-03, R&D Systems). Agarose was allowed to cool down for 20 minutes at 4°C. Then, cell membranes were permeabilized with a lysis solution (2.5 M NaCl, 100 mM EDTA, 10 mM Tris-HCl, 1% Triton X-100 [pH 10]) at 4°C for 1 hour. Slides were then equilibrated for 20 minutes in electrophoresis buffer (pH 12.3: 2 mM EDTA, pH adjusted to 12.3 with NaOH) at 4°C. Then, an electrophoresis field of 2.06 V/cm (98 V and approximatively 176 mA in an electrophoretic system where electrodes are 47.5 cm apart) was applied for 5 minutes at 4°C for RPTEC/hTERT1 cells, or for 3 minutes 30 seconds for H1975 cells. The electrophoretic migration was stopped by neutralizing the pH in a bath of cold water for 10 minutes. DNA was stained with SYBR Green (S7563, Invitrogen, Thermo Fisher Scientific)for 20 minutes at room temperature, according to the manufacturer’s recommendation. The slides were photographed under an Axio Imager Z1 Apotome microscope (Zeiss). The images were analyzed using an ImageJ in-home macro, in which the head (the nucleus) and tail (the DNA that migrated) of the comet were delimited to get the fluorescence intensity of the head, the fluorescence intensity of the tail, and the length of the tail. The calculation of tail moments was done using the following formula: (length of the comet tail × fluorescence intensity of the tail)/total fluorescence intensity (head + tail).

### Oil Red staining.

Frozen kidney mouse sections (10 μm) were fixed with ethanol (60%) and then incubated for 15 minutes with Oil Red O Solution (Fisher Biotec) dissolved in isopropanol. After several washes with ddH_2_O, samples were incubated for 3 minutes with hematoxylin. Lipid droplets were stained red, whereas nuclei appeared blue. RPTEC/hTERT1 cells were grown on coverslips in 24-well plates (75,000 cells/well) and exposed for 48 hours to cisplatin (50 μM) with or without 25 μM KW6002. RPTEC/hTERT1 cells were fixed with ethanol (60%) and then incubated for 15 minutes with Oil Red O Solution (Merck). The cells were washed 3 times with ddH_2_O and incubated for 3 minutes with hematoxylin. Coverslips were rinsed with H_2_0 before mounting on microscope slides using glycerol gelatin aqueous slide mounting medium (MilliporeSigma). Quantification was performed in a blinded manner using ImageJ software (NIH). Briefly, images were captured under light microscopy at ×400 magnification and processed using color deconvolution with RGB vectors. The resulting red color images were quantified using a custom threshold (0.173 for RPTEC/hTERT1 cells and 0.140 for kidney stainings).

### Immunofluorescence (cells).

LLC1 cells were cultured in Lab-tek (15,000 cells/well), and after 24 hours, the cells were exposed to cisplatin (2 μM) with or without 10 nM KW6002 for 6 hours. H1975 cells were cultured in Lab-tek (15,000 cells/well), and after 24 hours, the cells were exposed for 6 hours to cisplatin (50 μM) with or without 10 nM KW6002. Cells were fixed with 4% paraformaldehyde, permeabilized with DPBS/0.1% Triton X-100, and incubated first with anti-γH2AX (Ser139) antibody (1:400; no. 9718, Cell Signaling Technology) and then with an Alexa Fluor 488 secondary antibody (1:200; Life Technologies, Thermo Fisher Scientific). Samples were examined on an immunofluorescence microscope (Leica DMI8), and γH2AX nuclear foci were counted.

### Immunofluorescence (tissues).

Paraffin-embedded sections (3 μm thick) were deparaffinized with xylene and rehydrated in successive ethanol dilutions. Then, antigen retrieval was done by incubation in sub-boiling 10 mM sodium citrate buffer. Tissues were permeabilized in a 0.4% Triton X-100 solution, and nonspecific binding was blocked with a 5% BSA solution in TBS for 2 hours. Sections were then incubated overnight with an anti-γH2AX antibodies (1:50; no. 9718, Cell Signaling Technology). After washing, the secondary antibody (A10042) was incubated for 45 minutes at room temperature. Again, after washing, the nuclei were stained with a 300 nM DAPI solution (D1306, Life Technologies, Thermo Fisher Scientific). The slides were analyzed using a Zeiss LSM 880 confocal microscope. Quantification was performed using ImageJ.

For immunofluorescence studies in free-floating sections, mice were deeply anesthetized with pentobarbital sodium (50 mg/kg, i.p.) and then transcardially perfused with cold NaCl (0.9%) and 4% paraformaldehyde in PBS (pH 7.4). Kidneys were removed, post-fixed for 24 hours in 4% paraformaldehyde, and cryoprotected in 30% sucrose before being frozen at –40°C in isopentane (methyl-butane) and stored at –80°C. Longitudinal kidney sections (40 μm) were obtained using a Leica cryostat. Free-floating sections were stored in PBS-azide (0.2%) at 4°C. Longitudinal kidney sections were incubated with a donkey serum (D9663, MilliporeSigma) at 10% in PBS Triton X-100 (0.2%) for 1 hour and then incubated with anti-A_2A_R primary antibody (1:50; GP-Af1000, Frontiers Institute) for 72 hours at 4°C in Signal Boost (8114, Cell Signaling Technology). Alexa Fluor 568–conjugated secondary antibodies (1:500; Life Technologies, Thermo Fisher Scientific) were incubated overnight at room temperature. Lectin staining was performed by incubating sections for 1 hour at room temperature with *Lotus tetragonolobus* FITC conjugate (Vector Laboratories, FL-1321-2) diluted to 2 μg/mL in blocking medium. Sections were counterstained with DAPI (1:5,000; no. 62247, Thermo Fisher Scientific) and mounted on superfrost slides and left to dry. Then, they were covered with Vectashield Vibrance Antifade Mounting Medium (H-1700, Vector Laboratories). Images were acquired using a Zeiss LSM 710 confocal laser-scanning microscope at ×20 magnification. 3D reconstruction of confocal image stacks was performed using Imaris software (Bitplane).

### RNA extraction.

For renal, DRG, and spinal cord tissues, total RNA was extracted with phenol/chloroform and subsequently precipitated in isopropanol as described previously (76). Total RNA from cultured cells was extracted using an RNeasy Mini kit (QIAGEN) following the manufacturer’s instructions.

### RNA-Seq and analysis.

RNA-Seq libraries (*n* = 5–6/group) were generated from 500 ng total RNA using the Illumina TruSeq Stranded mRNA Library Prep Kit, version 2. Briefly, following purification with poly-T oligo attached magnetic beads, the mRNA was fragmented using divalent cations at 94°C for 2 minutes. The cleaved RNA fragments were copied into first-strand cDNA using reverse transcriptase and random primers. Strand specificity was achieved by replacing deoxythymidine triphosphate (dTTP) with deoxyuridine triphosphate (dUTP) during the second-strand cDNA synthesis by DNA polymerase I and RNase H (TruSeq Stranded mRNA, Illumina). Following the addition of a single “A” base and subsequent ligation of the adapter on double-stranded cDNA fragments, the products were purified and enriched with PCR [30 s at 98°C (10 s at 98°C, 30 s at 60°C, 30 s at 72°C) × 12 cycles; 5 min at 72°C] to create the cDNA library. Surplus PCR primers were further removed by purification using AMPure XP beads (Beckman Coulter), and the final cDNA libraries were checked for quality and quantified using capillary electrophoresis. Sequencing was performed on an Illumina HiSeq 4000 as single-end 50 base reads following Illumina’s instructions. Reads were mapped onto the mm10 assembly of the *Mus musculus* genome using STAR, version 2.5.3a (77). Only uniquely aligned reads were kept for further analyses. Quantification of gene expression was performed using HTSeq-count, version 0.6.1p1 (78), and gene annotations from Ensembl releases 90 and 102 and “union” mode. Read counts were normalized across libraries with the method proposed by Ander et al. (79). Comparisons of interest were performed using the test for differential expression proposed by Love et al. (80) and implemented in the DESeq2 Bioconductor library (version 1.16.1). The resulting *P* values were adjusted for multiple testing using the Benjamini-Hochberg method (81). RNA-Seq was performed by the Plateforme GenomEast, Institut de Génétique et de Biologie Moléculaire et Cellulaire, UMR 7104 CNRS-UdS/INSERM U964 (Illkirch). The sequencing data supporting the findings of this study have been deposited in the NCBI’s Gene Expression Omnibus (GEO) database (GEO GSE179247; https://www.ncbi.nlm.nih.gov/geo/query/acc.cgi?acc=GSE179247).

### Gene Ontology terms, STRING, and IPA.

Functional enrichment analysis was run with DAVID (Database for Annotation, Visualization and Integration Discovery; https://david-d.ncifcrf.gove/home.jsp), the STRING (Protein-Protein Interaction Networks; https://string-db.org/), or uploaded to the IPA web portal (QIAGEN; www.ingenuity.com). The data were analyzed to predict gene networks, molecular and cellular functions, canonical pathways, and upstream regulators of cisplatin and KW-modulated genes.

### GSEA analysis.

GSEA, version 4.1.0 (82, 83), was used, and a preranked analysis was run using the following settings: “No collapsing of gene symbols, the classic enrichment statistic, gene sets containing more than 500 genes and less than 50 genes were excluded from analysis” and with gene sets from Gene Ontology (GO). Genes were ranked on the basis of the values computed as follows: –10 × log_10_(*P* value) × fold change sense.

### Quantitative RT–PCR.

Reverse transcription was performed on 1 μg RNA using the High-capacity cDNA Reverse Transcription Kit (Thermo Fisher Scientific), according to the manufacturer’s instructions. Real-time PCR was performed on a StepOne device using TaqMan Gene Expression Master Mix (Thermo Fisher Scientific), according to the manufacturer’s recommendations. Expression levels of the following genes were evaluated using the comparative Ct method (2^–ΔCt^): *NGAL* (assay IDs Hs00194353_m1 and Mm01324470_m1); *KIM1* (assay IDs Hs00273334_m1 and Mm00506686_m1); *Tnfa* (assay IDs Hs00174128_m1 and Mm00443258_m1); *Il6* (assay IDs Hs00174131_m1 and Mm00446190_m1); adora2A receptor (*A2AR*, assay IDs Hs00169123_m1 and Mm00802075_m1); *Bax* (assay IDs Hs00180269_m1 and Mm00432051_m1); *Bcl2* (assay IDs Hs00608023_m1 and Mm00477631_m1); *MATE1/2* (assay IDs Hs00979028_m1/Hs00945652_m1 and assay IDs Mm00840361_m1/Mm02601002_m1); ATP-binding cassette subfamily C member 2 (*Abcc2*, assay IDs Hs00166123_m1 and Mm00496899_m1); nuclear factor erythroid–derived 2–like 2 (*NRF2*, assay IDs Hs00232352_m1 and Mm00477784_m1); heme oxygenase 1 (*HO1*, assay IDs Hs01110250_m1 and Mm00516005_m1); *Il1b* (assay ID Mm00434228_m1); chemokine (C-C motif) ligand 2 (*Ccl2*, assay ID Mm00441242_m1); selectin, platelet (*Selp*, assay ID Mm00441295_m1); complement component 3 (*C3*, assay ID Mm01232779_m1); chemokine (C-X-C motif) ligand 10 (*Cxcl10*, assay ID Mm00445235_m1); chemokine (C-X-C motif) ligand 12 (*Cxcl12*, assay ID Mm00445553_m1); *Tnf* (assay ID, Mm00443258_m1); and *Il6* (assay ID Mm00446190_m1). Transcript levels of *PPIA* (human Hs99999904_m1and mouse sample Mm02342430_m1) were used as an endogenous control.

### Renal tissue and cell concentrations of platinum.

RPTEC/hTERT1 cells were cultured in 6-well plates (250,000 cells/well) and exposed to cisplatin (50 μM) with or without KW6002 (25 μM) for 48 hours. H1975 cells were cultured in 6-well plates (150,000 cells/well) and exposed to cisplatin (20 μM) with or without KW6002 (10 nM) for 24 hours. Tissues (35 mg) and cell pellets were first mineralized with hydrochloric acid (30 % Suprapur, Merck) and nitric acid (69.5 %, Carlo Erba), respectively. Analysis of tissue samples was performed by Graphite Furnace Atomic Absorption Spectrometry (GF-AAS) using an AAnalyst 800 (Perkin Elmer), and cell sample analysis was performed by Inductively Coupled Plasma Mass Spectrometry (ICP-MS) using an ICAP-Qc (Thermo Fisher Scientific). The platinum concentration was finally normalized to the accurately measured kidney mass or to the protein concentration of the previously assessed cell lysates.

### Immunoblotting.

Cells and tissues were homogenized using RIPA buffer (MilliporeSigma) supplemented with protease and phosphatase inhibitors (Pierce, Thermo Fisher Scientific). Total protein (10 μg) was heated for 10 minutes at 70°C and loaded onto NuPAGE Novex gels (Thermo Fisher Scientific). After transferring the proteins onto nitrocellulose membranes, the membranes were blocked with 5% milk in TBS-Tween followed by incubation with anti–cleaved caspase 3 (1:1,000; no. 9661, Cell Signaling Technology); anti–cleaved PARP1 (1:1,000; no. 5625, Cell Signaling Technology); anti–cyclin D1 (1:1,000; sc-718, Santa Cruz Biotechnology); or anti-PCNA (1:1,000; no. 13110, Cell Signaling Technology) primary antibodies. Visualization of proteins was achieved using HRP-coupled secondary antibodies (1:2,000; no. 7074, Cell Signaling Technology). Signal detection was performed using the ECL Select Chemiluminescence Kit and ImageQuant LAS 4000 (both from GE Healthcare). Data were analyzed with ImageJ. Membranes were probed with anti-GAPDH (G9545, MilliporeSigma) or anti–β-actin (4970, Cell Signaling Technology) antibodies as normalizers.

### Statistics.

All data are presented as the mean ± SEM. Differences between groups were assessed using a 2-tailed Student’s *t* test, 1-way ANOVA followed by a multiple-comparison Tukey’s post hoc test, or repeated-measures 2-way ANOVA using GraphPad Prism (GraphPad Software). Differences were considered statistically significant at a *P* value of less than 0.05. The number of biologically independent experiments, sample size, *P* values, and statistical tests are all indicated in the main text or figure legends.

### Study approval.

All animal experiments were conducted in accordance with the European animal welfare regulation and US NIH guidelines on the ethical care of animals and were approved by the IACUCs of the University of Lille (protocol no. CEEA 2018101215473925) and Michigan State University.

## Author contributions

DB, GL, CC, FG, NP, and MP conceptualized the study. ED, MP, DB, CC, and GL designed the methodology. ED, KC, N Martin, N Massri, SF, JS, NB, RC, HR, CVDH, JVS, N Martin, N Massri, AL, JKF, CDS, RL, IL, MG, VGM, EF, JL, N Boukrout, N Beauval, and PM performed the experiments. ED, KC, MJ, CVDH, N Martin, N Boukrout, SLG, AG, VG, JBG, and JSA performed data analysis. GL, DB, and CC supervised the study. CC, DB, GL, JSA, and RAC wrote the original draft of the manuscript. KC, NP, CVDH, MP, LB, CA, FG, RAC, GL, DB, and CC wrote, reviewed, and edited the manuscript.

## Supplementary Material

Supplemental data

Supplemental table 1

Supplemental table 2

Supplemental table 3

Supplemental table 4

Supplemental table 5

Supplemental table 6

Supplemental table 7

Supplemental table 8

## Version 1 11/15/2022

Electronic publication
