# Supplementary material for: Istradefylline protects from cisplatin-induced nephrotoxicity and peripheral neuropathy while preserving cisplatin antitumor effects
Source: J Clin Invest. 2022 Nov 15;132(22):e152924. doi: 10.1172/JCI152924 (PMC9663157; doi:10.1172/JCI152924)
Supplement: Supplemental data [file jci-132-152924-s126.pdf]

Supplementary Materials for

Istradefylline (KW6002) protects from cisplatin-induced nephrotoxicity and peripheral neuropathy while preserving cisplatin anti-tumor effects

Edmone Dewaeles[1]†, Kévin Carvalho[2,3]†, Sandy Fellah[1]†, Jaewon Sim[4], Nihad Boukrout[1] Raphaelle Caillierez[2,3], Hariharan Ramakrishnan[4], Cynthia Van der Hauwaert[1,5], Jhenkruthi Vijayashankara[2,3], Nathalie Martin[1] Noura Massri[4] Agathe Launay[2,3], Joseph K. Folger[4], Clémentine De Schutter[1], Romain Larrue[1, 6], Ingrid Loison[1], Marine Goujon[1], Matthieu Jung[7], Stéphanie Le Gras[7], Victoria Gomez-Murcia[2,3], Emilie Faivre[2,3], Julie Lemaire[1], Anne Garat[6,8], Nicolas Beauval[6,8], Patrice Maboudou[9], Viviane Gnemmi[1,10], Jean-Baptiste Gibier[1,10], Luc Buée[2,3], Corinne Abbadie[1], Francois Glowacki[1,11], Nicolas Pottier[1,6], Michael Perrais[1], Rodrigo A. Cunha[12,13], Jean-Sébastien Annicotte[14,15], Geoffroy Laumet[4]‡, David Blum[2,3]‡, Christelle Cauffiez[1]‡

# Correspondance to:
David Blum, Lille Neuroscience & Cognition, Inserm UMR-S1172, "Alzheimer & Tauopathies", Place de Verdun, 59045, Lille Cedex, France. Orcid Number: 0000-0001-5691-431X. david.blum@inserm.fr.
Christelle Cauffiez, UMR9020 CNRS – UMR1277 Inserm, 1, place de Verdun ; 59045 Lille Cedex, France. Orcid : 0000-0003-0951-7973. christelle.cauffiez@univ-lille.fr.

This PDF file includes Figs. S1 to S4 and legends for Supplementary tables.

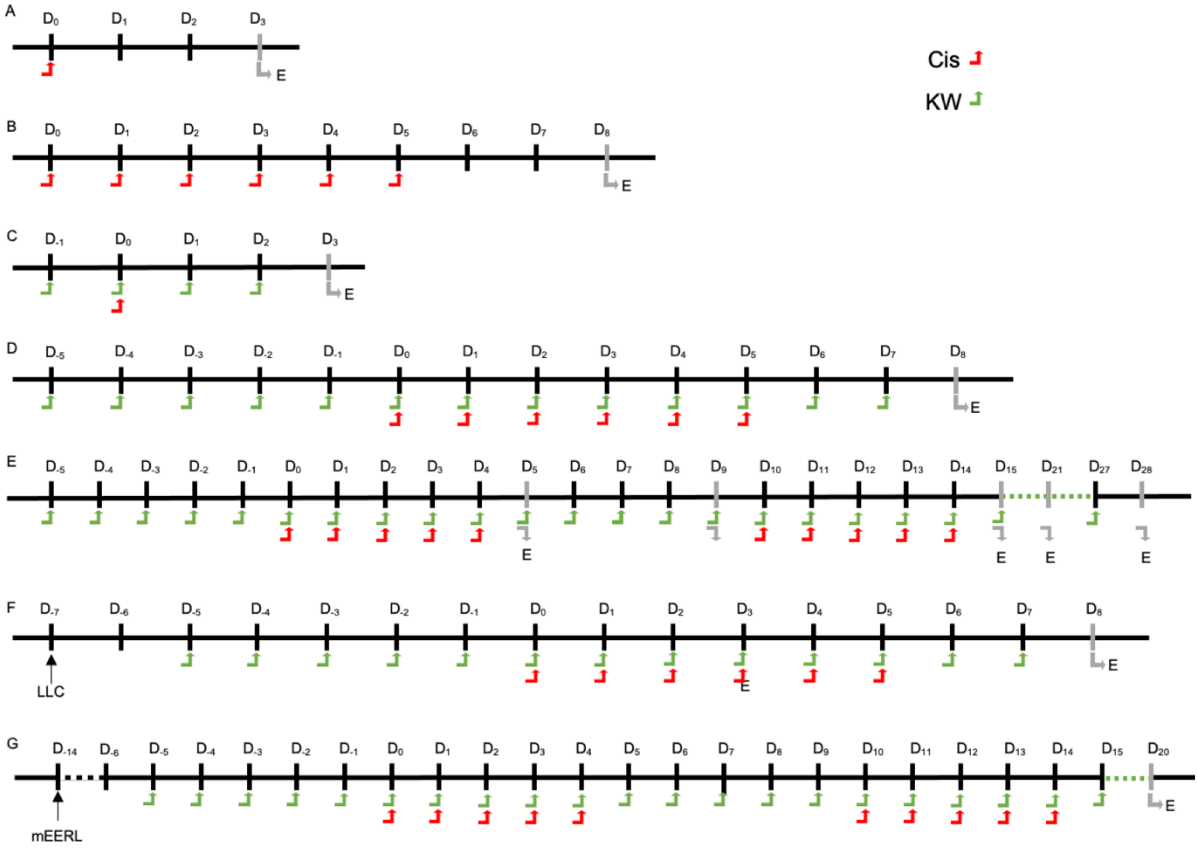

Figure S1. Schematic representation of the different animal procedures. (A) Acute cisplatin-induced kidney injury. C57BL6/J 8-weeks old male mice were administered an intra- peritoneal single injection of 10 mg/kg cisplatin (cis) and were sacrificed (E) three days (D) post-injection. **(B)** Sub-chronic cisplatin-induced kidney injury. C57BL6/J 8-weeks old male mice were intraperitoneally injected daily with cisplatin (3 mg/kg) for 6 days and were sacrificed 72 h after the last injection. **(C)** KW6002 administration schedule in the acute model. C57BL6/J 8-weeks old male mice were randomized to 4 groups: Vehicle, KW6002, Cisplatin or KW6002/Cisplatin. Cisplatin administration was performed as indicated in A. The first administration of KW6002 (3 mg/kg) was performed one day prior cisplatin treatment and daily until the sacrifice. **(D)** KW6002 administration schedule in the sub-chronic model. C57BL6/J 8-weeks old male mice were randomized to 4 groups: Vehicle, KW6002, Cisplatin or KW6002/Cisplatin. Cisplatin administration was performed as indicated in B. The first administration of KW6002 (3 mg/kg) was performed five days prior cisplatin treatment and daily until the sacrifice. **(E)** KW6002 administration schedule in the cumulative chronic model. C57BL6/J 8-weeks old male mice were randomized to 4 groups: Vehicle, KW6002, Cisplatin or KW6002/Cisplatin. Cisplatin administration was performed from D0-D4 and from D10-D14. The first administration of KW6002 (3 mg/kg) was performed five days prior cisplatin treatment and daily until the last sacrifice at D28. **(F)** Effects of KW6002 in the LLC1 syngeneic tumor mouse model treated with sub-chronic cisplatin. KW6002 and cisplatin administration were as performed in D. LLC1 cells (1 million) in PBS:matrigel (1:1, for a total volume of 100 µL) were subcutaneously injected in the right flank of all animals. When tumor volume reached 100 mm^3, mice were randomized into 4 groups: Vehicle, KW6002, Cisplatin or KW6002/Cisplatin. **(G)** Effects of KW6002 in the mEERL syngeneic tumor mouse model. KW6002 and cisplatin administration were as performed in E. mEERL cells (1, 000,000) were subcutaneously injected in the right flank. When tumor volume reached 100 mm^3, mice were randomized into 4 groups: Vehicle, KW6002, Cisplatin or KW6002/Cisplatin.

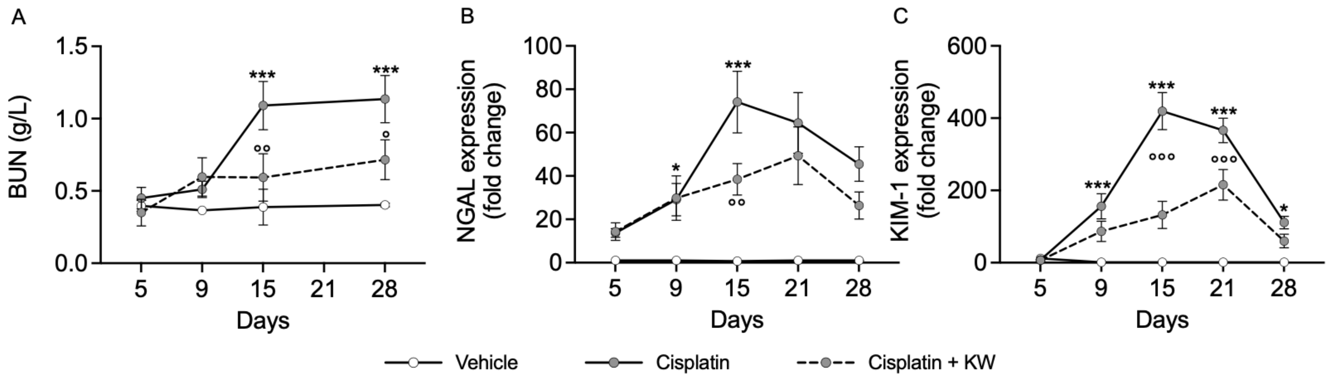

Figure S2. KW6002 protects from cisplatin-induced kidney injury in a cumulative model of cisplatin toxicity. (A) BUN quantification. **(B-C)** Gene expression of the renal injury markers *NGAL* **(B)** and *KIM-1* **(C)**. Data are mean ± SEM. *p<0.05, ***p<0.001 vs. Vehicle; °p<0.05, °°p<0.01, °°°p<0.001 vs. cisplatin (n=6 animals/group; Two-Way ANOVA followed by a Tukey's post-hoc test).

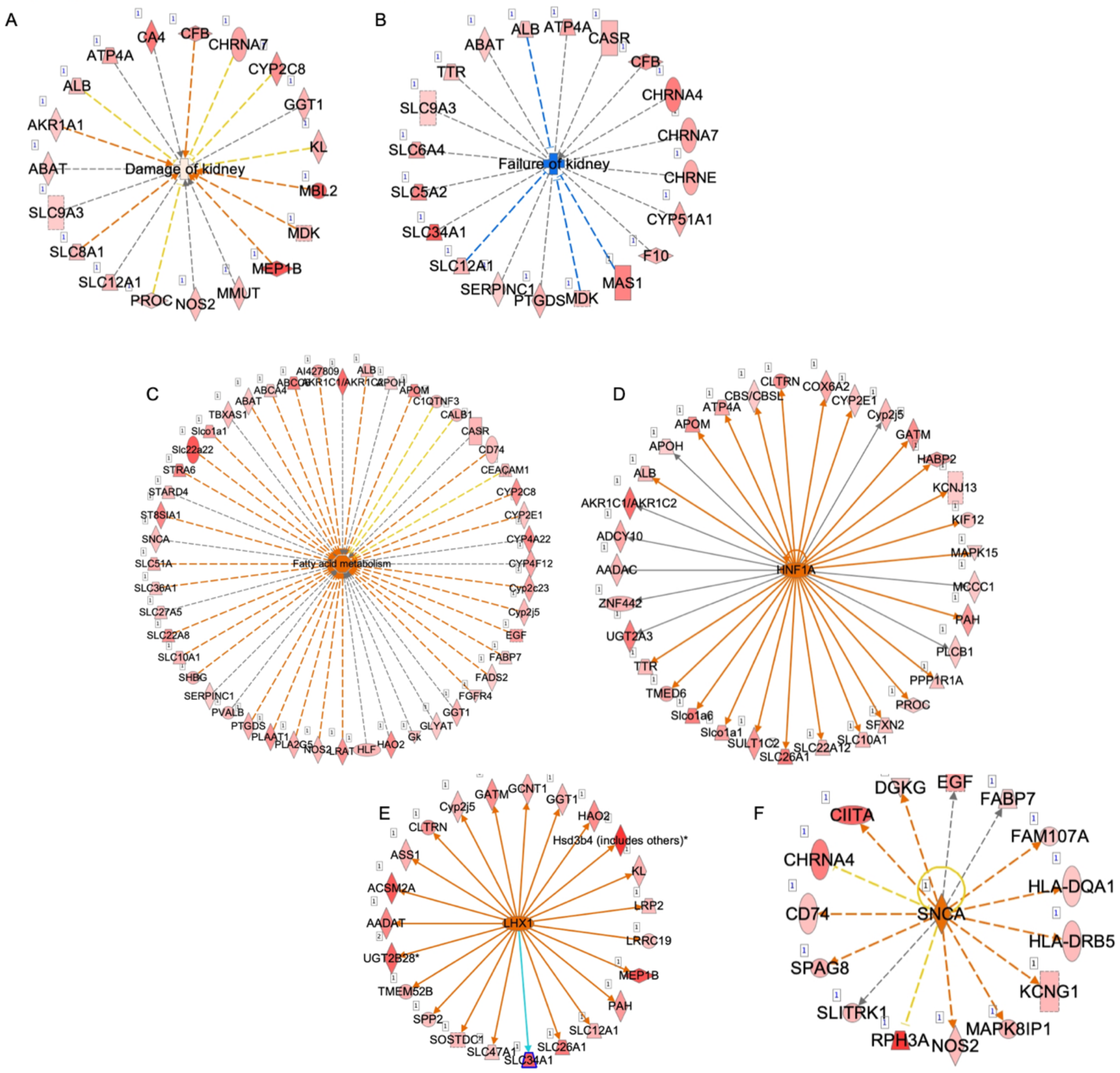

Figure S3. Ingenuity Pathway Analysis (IPA) identified significant gene networks and upstream regulators associated to "damage of kidney", "failure of kidney" and "fatty acid metabolism". The 635 downregulated by cisplatin and normalized by KW6002 (Figure 4, G-I) were analyzed using IPA and effects were predicted for **(A)** damage and **(B)** failure of kidney, and **(C)** fatty acid metabolism. Genes from the sequencing are in red (upregulated), while predicted functions and interaction lines are in blue (inhibition) or orange (activation). Yellow lines indicate inconsistent findings, and gray dotted lines show unpredicted effects. Using IPA, upstream regulator analysis predicted activation of HNF1a **(D)**, LHX1 **(E)** and SNCA **(F).**

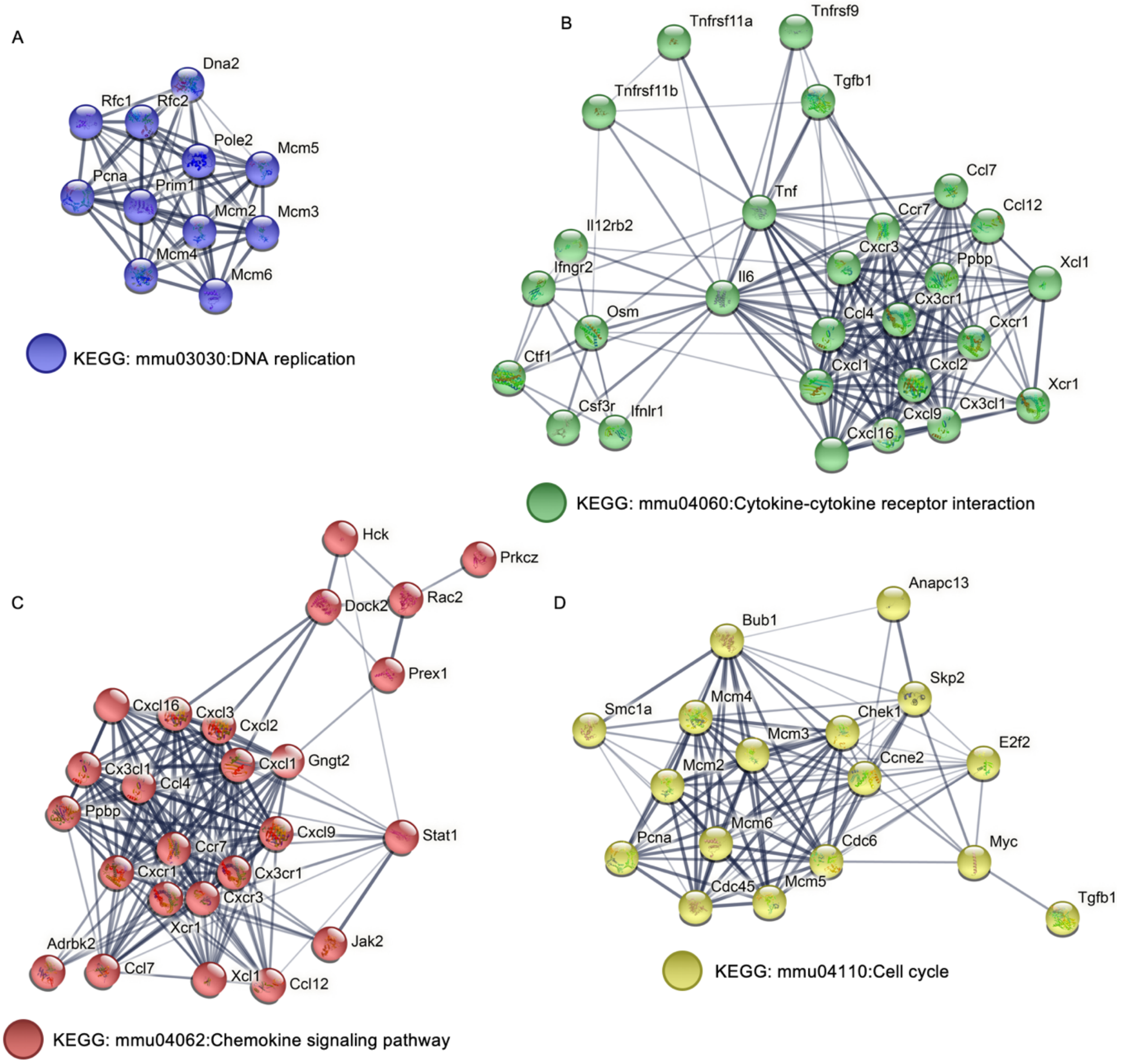

Figure S4. STRING networks from the four most significant KEGG pathway enrichment of the 1016 genes that are only downregulated (see Figure 6H, red) by KW6002 in the context of cisplatin co-treatment. **(A)** DNA replication, **(B)** Chemokine signaling pathway, **(C)** Cytokine-cytokine receptor interaction, **(D)** Cell cycle.

Supplementary Tables

Table S1. Relative expression of inflammatory molecules in kidney, DRG and spinal cord samples from the sub-chronic cisplatin administration model. mRNA levels were evaluated by qPCR. Data are mean ± SEM. *p<0.05, **p<0.01, ***p<0.001 vs. Vehicle; °p<0.05, °°p<0.01, °°°p<0.001 vs. cisplatin (n=5-6 animals/group; One-Way ANOVA followed by a Tukey's post-hoc test).

Table S2. Unsupervised GSEA analysis of pathways modulated by KW6002 in the kidney of cisplatin-treated animals. **(A)** Unsupervised GSEA analysis of pathways downregulated by KW6002 in the kidney of cisplatin-treated (NES<-5; FDR<1.73.10^{5}). **(B)** Unsupervised GSEA analysis of pathways upregulated by KW6002 in the kidney of cisplatin-treated animals (NES>5; FDR<1.25.10^{5}).

Table S3. Disease and biological functions affected by KW6002/cisplatin treatment.

Table S4. Toxicological functions affected by KW6002/cisplatin treatment.

Table S5. Upstream regulator analysis affected by KW6002/cisplatin treatment.

Table S6. Unsupervised GSEA analysis of pathways upregulated by KW6002 in LLC1 tumors of cisplatin-treated animals (NES<-4; FDR<3.04.10^{5}).

Table S7. Disease and biological functions affected by KW6002/cisplatin treatment.

Table S8. Gene networks affected by KW6002/cisplatin treatment.
