# Supplementary material for: Istradefylline protects from cisplatin-induced nephrotoxicity and peripheral neuropathy while preserving cisplatin antitumor effects
Source: J Clin Invest. 2022 Nov 15;132(22):e152924. doi: 10.1172/JCI152924 (PMC9663157; doi:10.1172/JCI152924)
Supplement: Supplemental table 1 [file jci-132-152924-s127.pdf]

Kidney

N=6	**Vehicle**	**KW**	**Cisplatin**	**Cisplatin + KW**
Il1b	1.00 ± 0.03	1.19 ± 0.10	1.34 ± 0.09$^{0.07}$	0.78 ± 0.12°°
Ccl2	1.00 ± 0.01	1.42 ± 0.20	8.20 ± 2.48**	2.38 ± 0.99°
Selp	1.00 ± 0.08	1.01 ± 0.12	1.07 ± 0.09	0.94 ± 0.14
C3	1.00 ± 0.18	1.32 ± 0.72	21.44 ± 6.42**	8.08 ± 2.29°
Cxcl10	1.00 ± 0.12	1.38 ± 0.12	9.11 ± 2.86*	2.53 ± 1.92°
Cxcl12	1.00 ± 0.08	1.34 ± 0.13	36.67 ± 8.63***	16.85 ± 6.70$^{0.07}$
Tnf	1.00 ± 0.08	1.26 ± 0.38	4.75 ± 0.79***	2.05 ± 0.54°°
Il6	1.00 ± 0.07	1.04 ± 0.20	22.67 ± 4.75***	9.46 ± 3.61°

DRG

N=5	**Vehicle**	**KW**	**Cisplatin**	**Cisplatin + KW**
Il1b	1.00 ± 0.08	0.99 ± 0.20	1.48 ± 0.31	0.81 ± 0.12°°
Ccl2	1.00 ± 0.15	0.91 ± 0.17	1.63 ± 0.23*	0.99 ± 0.17°°
Selp	1.00 ± 0.11	0.90 ± 0.27	1.39 ± 0.15	1.04 ± 0.10
C3	1.00 ± 0.11	0.89 ± 0.15	1.44 ± 0.33	0.70 ± 0.15°°°
Cxcl10	1.00 ± 0.13	0.46 ± 0.04	0.56 ± 0.16	0.72 ± 0.21
Cxcl12	1.00 ± 0.10	0.98 ± 0.22	1.00 ± 0.33	1.09 ± 0.09
Tnf	1.00 ± 0.16	0.64 ± 0.03	1.30 ± 0.36	0.65 ± 0.04°
Il6	1.00 ± 0.16	1.06 ± 0.22	1.37 ± 0.18	0.85 ± 0.01°

Spinal cord

N=5	**Vehicle**	**KW**	**Cisplatin**	**Cisplatin + KW**
Il1b	1.00 ± 0.07	0.77 ± 0.12	1.29 ± 0.19	1.13 ± 0.04
Ccl2	1.00 ± 0.18	1.09 ± 0.25	0.88 ± 0.28	1.46 ± 0.06
Selp	1.00 ± 0.12	0.95 ± 0.19	1.52 ± 0.16	1.37 ± 0.21
C3	1.00 ± 0.03	0.87 ± 0.17	1.47 ± 0.30	1.11 ± 0.10
Cxcl10	1.00 ± 0.13	1.07 ± 0.26	1.43 ± 0.21	0.85 ± 0.08
Cxcl12	1.00 ± 0.17	1.10 ± 0.18	0.90 ± 0.15	1.10 ± 0.00
Tnf	1.00 ± 0.12	0.99 ± 0.09	1.36 ± 0.22	0.87 ± 0.20°
Il6	1.00 ± 0.19	0.80 ± 0.08	1.03 ± 0.23	0.71 ± 0.07
